# Less is more: usefulness of data flow diagrams and large language models for security threat validation

**DOI:** 10.1007/s10664-026-10837-z

**Published:** 2026-04-21

**Authors:** Winnie Bahati Mbaka, Katja Tuma

**Affiliations:** 1 Vrije Universiteit Amsterdam, Amsterdam, The Netherlands; 2 Eindhoven University of Technology, Eindhoven, The Netherlands

**Keywords:** STRIDE, Data flow diagrams, Large language models, Threat validation, Empirical software engineering

## Abstract

The arrival of recent cybersecurity standards has raised the bar for security assessments in organizations, but existing techniques require a high manual effort. Threat analysis and risk assessment are used to identify security threats for new or refactored systems. Still, there is a lack of definition-of-done, so identified threats have to be validated which slows down the analysis. Existing literature has focused on the overall effectiveness of threat analysis, but no previous work has investigated what material must the analysts use to effectively validate the identified security threats. We conduct a controlled experiment with practitioners to investigate whether having some analysis material (either the system’s graphical model or LLM-generated advice) is better than none, and whether having both the system’s graphical model and LLM-generated advice is better than having only one of them. We run a pilot of the experiment with 41 MSc students, a think-aloud study with three practitioners, and the experiment survey with 68 recruited practitioners. Our main findings suggest that, in terms of additional material needed for threat validation, less is more. We also find that participants perceived the graphical model as equally useful compared to LLMs and that, despite LLMs not always providing conclusive advice, practitioners still perceived it as somewhat useful. The experimental material and data analysis scripts is publicly available in a replication package.

## Introduction

Building secure software is a global concern that has in recent years spurred new regulations (e.g., EU Cybersecurity and Cyberresilience Acts, and the US Cloud Act). CISA and 17 U.S. and international partners (Cisa et al. 2023) recommend planning for countermeasures to reduce the risk of costly security breaches later on. For safety-critical systems, such as systems developed in the transportation sector, recent standards even require conducting a threat analysis of entire products (ISO/SAE 21434:2021 ISO, SAE 21434:2021, 2021). In light of the global gap in the cybersecurity workforce, these demands can be disruptive for organizations.

Threat analysis and risk assessment methods help to elicit critical security threats and identify appropriate countermeasures (Tuma et al. 2018). STRIDE is a threat analysis methodology developed at Microsoft that guides the identification of six different types of security threats: Spoofing, Tampering, Repudiation, Information disclosure, Denial of services, and Elevation of privilege. This methodology is extensively used in practice, for instance, in the automotive industry (Macher et al. 2016), at Microsoft (Shostack 2014), and in agile organizations (Bernsmed and Jaatun 2019). STRIDE (Shostack 2014) uses a graphical representation of the software architecture under analysis, the *Data Flow Diagram* (DFD) (Deng et al. 2011; Sion et al. 2018), which is simple and easy to learn (Scandariato et al. 2015). DFDs are system models that provide a high-level architectural view of the software and graphically depict how information moves across system components (Tuma and Scandariato 2018). During a threat analysis session, security and domain experts explore the DFD (and the available material) to identify potential security threats through brainstorming (Shostack 2014). But this process is time-consuming (Scandariato et al. 2015), and the resources dedicated to security are scarce in organizations.

Without enough objective measures for threat correctness and completeness (Mbaka and Tuma 2023; Tuma et al. 2018; Soares Cruzes et al. 2018), predicting threats for software that has not been built yet demands practitioners to make risk-based decisions under uncertainty (Bier 2020). Previous work has found that practitioners often waste time revisiting the analysis material and *validating threat feasibility* (Tuma et al. 2021) to assert that they have done a "complete job" and that they have not overlooked any important security threat. While feasibility of some threats is quickly determined (e.g., SQL injection to an exposed web interface), more complex and domain-specific threats require a nuanced reasoning. In such cases, practitioners may have to revisit analysis material (such as the DFD, the requirements of the system, general or domain-specific security catalogues, such as MITRE ATT&CK knowledge base^1^).

Large Language Models (LLMs) are being used for providing security advice and summarizing information (Chen et al. 2023) and tools for using LLMs for security risk analysis are emerging (Adams 2024; Brejcha 2024; Esposito and Palagiano 2024). For example, a customized GPT (Brejcha 2024) that provides suggestions for prompts, which generate a list of potential security threats. Even if in-house models are deployed in a secure environment, LLMs tend to hallucinate (Huang et al. 2023), so the generated list of threats may be eventually used as *additional* analysis material to avoid overlooking threats, as opposed to removing the human altogether.

*So, what material must the analysts use to effectively validate security threats?*

We investigated the usefulness of analysis material for threat validation, with the goal of understanding if reading "some" additional analysis material is better than "none" and if "more" available analysis material is better than "some". Since practitioners tend to refer to (and revisit) the DFD during threat validation by assessing threat priority and feasibility (Tuma et al. 2021), it is interesting to include the presence of a DFD for this task as an intervention. In addition, having the DFD available could potentially help the practitioners in assessing the LLM advice, therefore it is also interesting to observe the presence of both DFD and LLM advice.

Due to known issues with hallucinations (Huang et al. 2023), we expect that if participants trust the first generated advice, this can increase the number of false positives. On the other hand, we expect that participants not fully trusting the first generated advice may perform better. Our results could inform practitioners about the minimal analysis material required for effective threat validation, and provide insights to practitioners that are on the fence about adopting LLMs for threat analysis.

This manuscript presents the results of the final phase of a Registered Report presented at the Empirical Software Engineering and Measurement (ESEM) conference (Bahati Mbaka and Tuma 2024). We conducted the planned study with 68 practitioners.

## Background

STRIDE is a threat analysis methodology developed at Microsoft to help identifying potential attack scenarios in the early stages of the Software Development Lifecycle (SDLC).

**Fig. 1:**
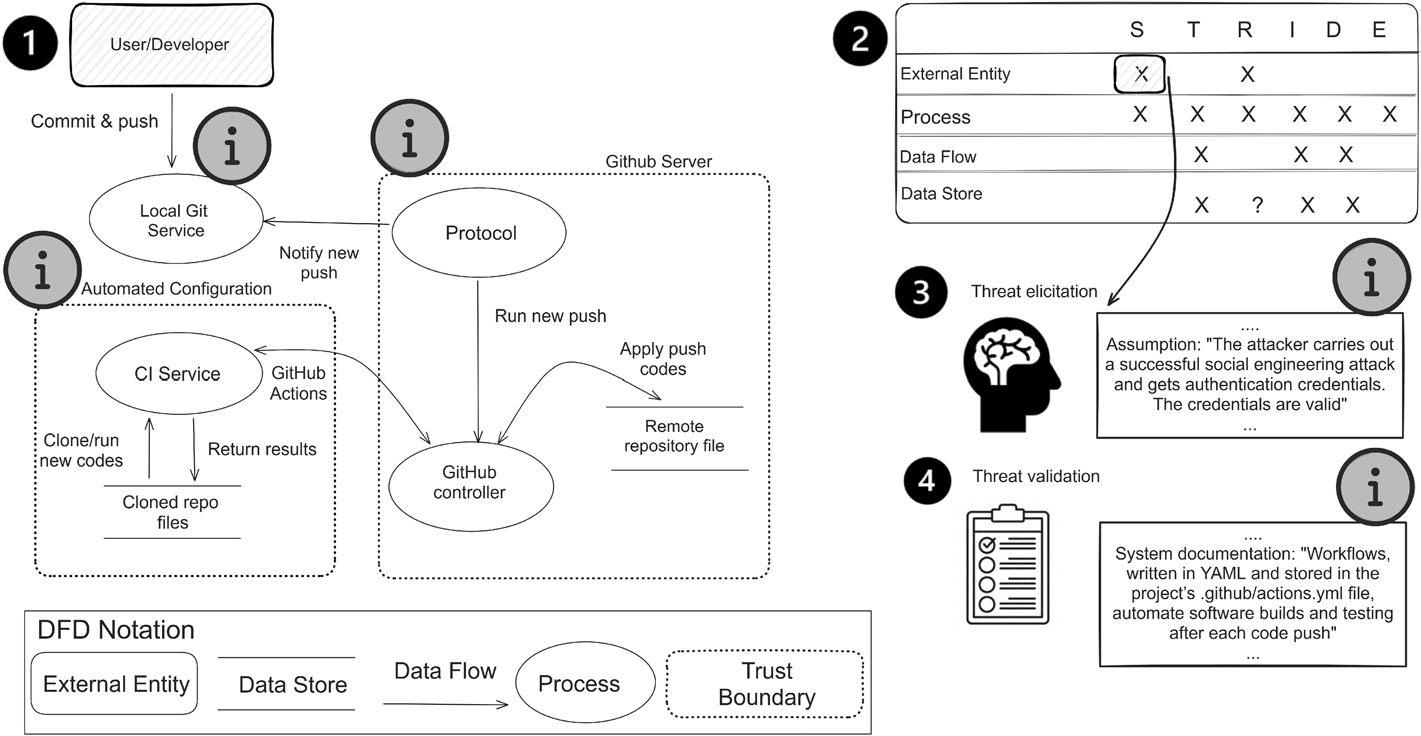
Overview of steps in threat analysis with STRIDE on a simplified DFD for the scenario of GitHub repository update. Information cues that might help in validating threat feasibility are marked with icon

Figure 1 presents an overview of the activities performed during a threat analysis with STRIDE. This technique is best applied after decomposing the system under analysis into its individual components (Khan et al. 2017; Möckel and Abdallah 2010), thereby creating a Data Flow Diagram (DFD). Step 1 in Fig. 1 shows a simplified DFD depicting the scenario of updating a remote repository on GitHub. During the analysis, security analysis and domain experts visit each element (or interaction) at a time and explore specific threat categories as defined by the mapping table, see step 2 in Fig. 1. For instance, for processes (e.g., GitHub controller), the threat-to-element mapping table suggests that analysts should investigate all six threat categories, while for external entities (e.g., User/Developer), the analysts are suggested to focus on spoofing and repudiation threats. Once all the elements have been visited and the specific threats identified, the analysts brainstorm and develop attack scenarios for each identified threat, step three in Fig. 1. The final step involves validating the feasibility of the identified threats and prioritizing them based on risk, step 4. Various *information cues* () are present in the analysis material and could help validating threats.

The usefulness of DFDs in understanding security risks is already well understood (Labunets et al. 2017). However, their actual and perceived effectiveness in threat validation is still an open question. In our study, we presupposed that DFD could help in threat validation by highlighting the presence or absence of critical security information. For instance, the lack (and presence) of trust boundaries. Sion et al. (2020) outline the various interpretations of having a trust boundary. First, trust boundaries represent different levels of privileges, second, they can be used to infer assumptions on the attacker model, and lastly, they can be used as a source of deployment-specific information. Given our DFD from Fig. 1, the presence of trust boundaries around the remote GitHub server and the CI service () indicates that specific privileges are required to interact with these components. On the other hand, the absence of one around the local git service () can be interpreted as a point of vulnerability where threats such as spoofing or information disclosure can occur.

Textual descriptions documented during the analysts’ brainstorming session to elicit threats (step 3) and any other available system documentation could also help to validate the existence or absence of specific threats (step 4). These textual descriptions provide insights into the attacker behaviour, system architecture, or design constraints that should be taken into consideration when validating threats. For instance, documented assumptions in step 3 establish preconditions that make spoofing feasible (), while step 4, offers insights into how CI/CD (continuous integration/continuous deployment) processes are triggered (). Such information cues help determine whether an elicited threat is technically feasible within the system’s actual configuration. Finally, the LLM response affirming that a security threat is feasible is another information cue that could help make the decision (not depicted in Fig. 1). For a complete overview of information cues in the analysis materials for each security threat, see Appendix A.3.

Beyond the study’s objectives, the use of DFD was included not only as an analytical tool to assist in threat validation but also as a complementary graphical representation of the textual materials (scenario description). The decision to have both graphical and textual descriptions was a choice in the study design to control for any cognitive variance in how individuals process information. Moreover, related work has investigated the actual and perceived efficacy of presenting both graphical and textual materials in understanding security risk. Labunets et al. (2017) found that participants using graphical models to conduct a security risk assessment perform equally in terms of actual and perceived efficacy compared to participants using textual information. Yet, participants perceived that graphical models were more useful compared to participant perception of textual information.

## Related Work

We positioned our contributions with respect to the existing literature on empirical studies of threat analysis, the application of the think-aloud protocol in software engineering, and Large Language Models in security.

### Empirical Research of Threat Analysis

Several studies (Scandariato et al. 2015; Mbaka and Tuma 2023) have empirically investigated the effectiveness of threat analysis in controlled settings with student participants. Both Scandariato et al. (2015) and Mbaka and Tuma (2023) replicated a controlled experiments that sought to measure the productivity, precision, and recall of STRIDE. The main findings of the first study (Scandariato et al. 2015) indicate that the methodology had a relatively high time cost, but its application was not perceived as difficult. In addition, while the number of incorrect threats was low (high precision), the number of overlooked threats was high (low recall) (Scandariato et al. 2015).

Mbaka and Tuma (2023) compared the productivity and precision of two STRIDE techniques, per-element and per-interaction. In the case of Mbaka and Tuma (2023) STRIDE-per-interaction teams performed better than their counterparts. However, no significant location shift between the productivity and precision of the two variants was reported.

Beyond measuring the effectiveness of STRIDE, other studies have investigated the challenges of adopting STRIDE in agile software development (Soares Cruzes et al. 2018; Bernsmed and Jaatun 2019). Bernsmed and Jaatun (2019) conducted interviews with employees from four organizations that use agile software development practices and observed several challenges: lack of motivation exhibited by the tendency of developers to skip threat modelling given a chance, difficulties in identifying relevant threats due to either a lack of experience or detailed guidelines, threat modelling was considered to be time-consuming, and none of the organisations had a clear definition of being "done" (i.e., when threat modelling is considered to be complete). Some of these challenges were also observed in Soares Cruzes et al. (2018); Galvez and Gurses (2018). Despite these shortcomings, Bernsmed and Jaatun (2019) reported that practitioners were in agreement that performing a threat analysis leads to a more secure product.

Apart from STRIDE, other threat analysis techniques, have been empirically investigated. Two studies have compared the effectiveness of attack trees and misuse cases (Karpati et al. 2014; Andreas et al. 2009). In the case of Karpati and colleagues (Karpati et al. 2014), industry practitioners were tasked with applying each technique (attack trees and misuse cases) on two different systems, leading to a within-subject experimental approach. The authors observed that attack trees resulted in the identification of a higher number of threats compared to misuse cases, albeit the difference was not statistically significant. Although the experimental set-up implemented by Andreas et al. (2009) was different from that of Karpati et al. (2014) (student participants vs industry practitioners) similar observations of better effectiveness when using attack trees were also reported in the former Andreas et al. (2009).

On the other hand, Mamadou et al. (2006) compared the applicability of three techniques (i.e., the common criteria, attack trees, and misuse case) applied to wireless hotspots. The study aimed to evaluate these techniques based on their learnability, usability, inclusiveness of solutions or mitigation, output clarity, and analysability. The authors observed that each technique has strengths and weaknesses. For instance, while common criteria were complex to learn, they were easy to analyse. On the other hand, misuse cases had easier learnability, but the output was not easy to read. Lastly, attack trees produce clear output, but are difficult to analyse (Mamadou et al. 2006).
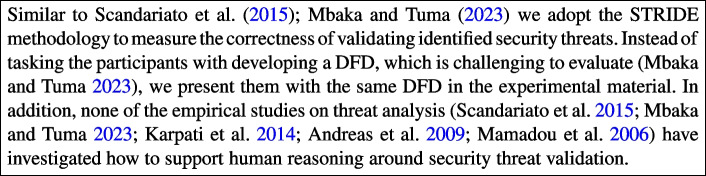


### Think-Aloud Protocol in Software Engineering

Almost 15 years ago, empirical studies involving human participants formed less than 2% of research in software engineering (Runeson and Höst 2009). Empirical investigations rely on various data collection methodologies. Among them is the think-aloud protocol, which requires participants to express their thought processes while undertaking a certain task (Seaman 1999).

Several studies have implemented the think-aloud protocol in secure practices (Votipka et al. 2020; Thompson et al. 2018; Wijayarathna and Arachchilage 2019) and software development (Gopstein et al. 2020). For example, Votipka and colleagues (Votipka et al. 2020) presented a validated scale measuring the self-efficacy of secure software development (SSD-SES). The authors used the think-aloud protocol as a qualitative pilot by asking practitioners in secure development to review their initial set of scale items. The resulting scale from the refinement with practitioners was further improved using a think-aloud protocol with a sample of their target population, software developers.
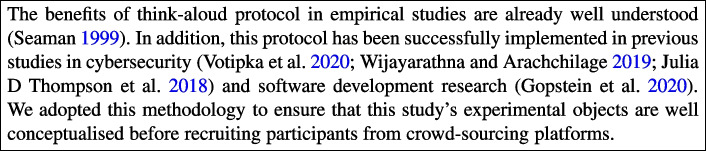


### Application of LLMs in Security

Several studies have evaluated the usefulness of LLMs in the detection of code and software vulnerabilities (Thapa et al. 2022; Omar 2023; Sun et al. 2023; Cheshkov et al. 2023). For instance, Omar (2023) proposes a vulnerability detection framework, VulDetect leveraging the strengths of three models (GPT-2, BERT, and LSTM) to detect C and C++ source code vulnerabilities. The authors observed that VulDetect was able to outperform other state-of-the-art vulnerability detection techniques (SyseVR and VulDeBert) with a 92.65% accuracy.

Cheshkov et al. (2023) sought to investigate the effectiveness of large language models (ChatGPT and GPT-3) in vulnerability detection. The study utilised datasets of Java files that contained both vulnerable and patched code obtained from open GitHub repositories. The authors compared the results of their models of choice to that of a baseline dummy classifier.

In the study by Sun and colleagues (2023) GPTScan (a combination of GPT and static analysis) was proposed and used to detect logic vulnerabilities in smart contracts. The authors further subdivided each logic vulnerability type into scenarios (describing the code functionality where the logic vulnerability would occur) and properties (the vulnerable code attributes), which enabled them to leverage GPT’s code understandability capabilities (Sun et al. 2023).

Chen and colleagues (2023) investigated the performance of two LLMs (ChatGPT and Bard) to refute popular security and privacy misconceptions. The authors curated a dataset containing 122 security and privacy misconceptions from existing literature. The study reported that both models had a correctness rate of above 70%, that is, they were able to correctly negate majority of the misconceptions (Chen et al. 2023).
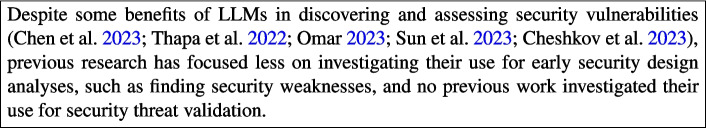


## Research Questions

Our research is motivated by previous findings pointing to the challenges with threat analysis reproducibility (Mbaka and Tuma 2023) and lack of definition-of-done (Soares Cruzes et al. 2018; Galvez and Gurses 2018). A significant challenge is determining the feasibility of the identified threats, or in other words, validating the identified threats, an activity that can take place several times during the process and has been measured to cause detours and slow down the progress (Tuma et al. 2021; Iqbal et al. 2020). Threat validation is not only an issue at the design phase, but also during threat intelligence gathering (Chadni Islam et al. 2022), where the collected data is also tainted by uncertainty.

On the other hand, previous research has investigated the effectiveness of providing additional textual or graphical material to aid in the comprehension of functional requirements (Abrahao et al. 2012) and safety compliance needs (Luis et al. 2020). We postulate that understanding what material (if any) should be used to effectively validate threats may help in deriving threat feasibility faster.

To this end, we formulate the first research question:

**RQ1: What is the actual usefulness of having additional material like DFD or LLMs during threat validation?**

To measure the actual usefulness of having additional supporting materials (DFDs, LLMs, or both), we define several treatment groups. First, we consider the actual effectiveness of participants validating threats without any additional material. To this end, we first compare their actual effectiveness to those who received some (a DFD, or an LLM). Second, we compare the actual effectiveness of those who received some material to those who received both a DFD and LLM. We hypothesize that actual usefulness should be different between groups that did not receive additional materials to those that did. That is, having some additional material (a DFD, or LLM) raises the actual effectiveness of participants. In addition, having both a DFD and LMM should increase the participant’s actual effectiveness to correctly identify realistic threats. We therefore propose the following alternative hypothesis;

$H_{actual-eff}$: *There is a statistically significant difference in the actual usefulness (i) between participants assessing the validity of threats without additional materials to those with some (either a DFD, or an LLM) and (ii) between participants assessing the validity of threats with some additional materials (DFD or LMM) to those with both (DFD and LLM).*

Second, some previous research measured differences (Liu et al. 2021) in the perceived usefulness of graphical models compared to textual information for security risk assessment. But, Labunets et al. (2017) found that tabular and graphical methods are statistically equivalent to each other with respect to the actual and perceived efficacy. Following up on this result, we also measure perceived usefulness of the material provided to support threat validation and expect to confirm equivalence in our study.

**RQ2: What is the perceived usefulness of the additional material during threat validation?**

To this end, we check for statistical equivalence using the alternative hypothesis formulated below;

$H_{equiv-perc-both}$: *When given both the DFD and LLM, their perceived usefulness is statistically equivalent.*

Second, the sample means of the treatment group that received only the DFD is compared to the one asked to assess the correctness of threats using only LLM. To this end, we formulate an alternative hypotheses;

$H_{equiv-perc-isolation}$: *The perceived usefulness of DFDs and LLMs when used in isolation is statistically equivalent.*

## Methodology

This section presents the design of the experiment, starting with an overview of the methods used in the work.

### Overview of Mixed-method Empirical Approach and Data Collection

**Fig. 2:**
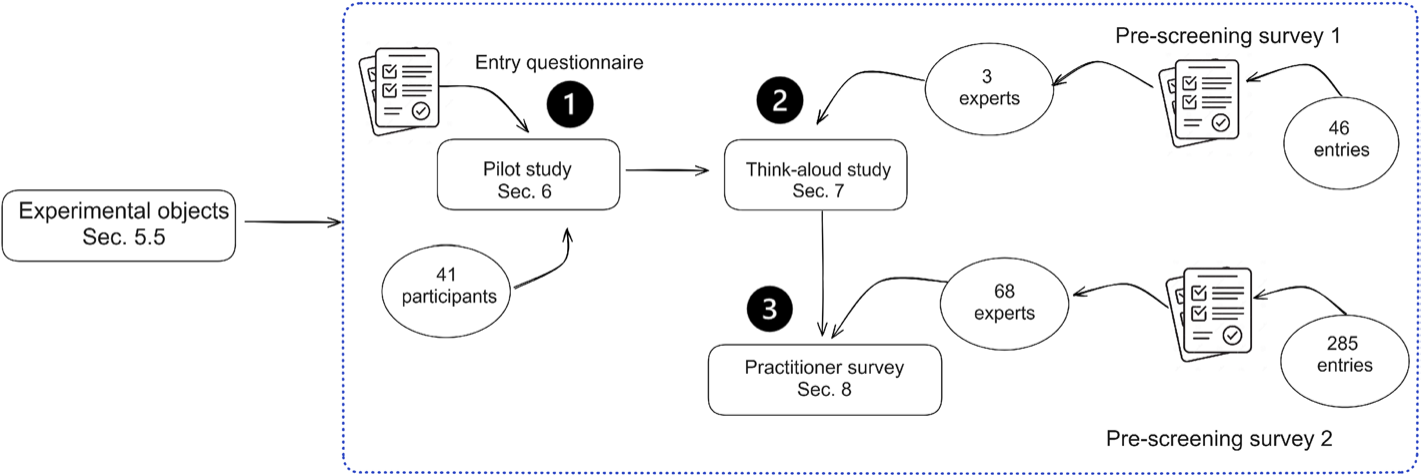
Overview of the mixed-method empirical approach

Figure 2 presents an overview of the mixed-method used and the number of finally recruited participants. We use the experimental design and material from Subsections 5.4 and 5.5, respectively, in three studies: a quantitative pilot study with students, a qualitative think-aloud study, and a quantitative survey with practitioners recruited from Upwork. While we use the same experimental objects in all the studies, the recruitment process differed. We first present the participant recruitment process, and ethical concerns. Then we continue to describe the experimental design, experimental objects, and the data analysis plan.

### Participant Recruitment

*Pilot study with students.* We define two target populations for this study. First, for the quantitative pilot study, we recruited Master Computer Science students attending courses taught by the experimenters. To ensure that the students understand the experimental objects necessary to conduct the study we conducted a 4h30min training, evaluated their understanding of the training material, and included attention checks to filter dishonest responses.

*Studies with practitioners.* We conducted the study with practitioners recruited from the Upwork crowd-sourcing platform. The purpose for performing the think-aloud was to make sure the study material and the experimental setting was polished and ready for deployment to practitioners on Upwork. During the sessions, participants were asked to complete the task with the survey instrument. They were also asked to verbalise their thoughts and decision-making processes while completing the questionnaire.

We recruited three practitioners for the qualitative, think-aloud pilot study. We pre-screened and recruited participants with a background in software development, cybersecurity risk management. Since threat modeling is carried out by domain experts, such as developers, software architects, and security specialists (Soares Cruzes et al. 2018), we consider such a sample as representative.

For the practitioner survey, all interested participants were required to complete a pre-screening survey. The pre-screening survey contained 20 multiple-choice questions (10 for each scenario used- GitHub and Kubernetes) used to gauge their understanding of the experimental objects.

For both participants recruited for the think-aloud study and practitioner survey, they were informed that only eligible candidates would be invited to participate in the full study and receive compensation. Only participants that were selected with the pre-screening surveys and actually participated in the study were compensated. Participants in the think-aloud additionally received $100 while those recruited in the practitioner survey received $90 each.

### Ethical Concerns

This experiment received ethical approval from the ethical board of the institution under review number 2024-013. First, the study provides an opt-in consent ("yes"/"no") form. Second, we do not anticipate any potential risk to the participants or researchers. Third, the study utilises GDPR-compliant tools to collect data. In addition, any personally identifiable information (PII) are removed before data analysis. Fourth, for the pilot with students, the study was conducted as part of a course, and participants were only incentivised with a participation point which has a very small effect on their final grade. Importantly, students who did not provide consent for the analysis of their data also received a participation point. The participants were debriefed about the process of the experiment and the artefacts used.

Finally, our study with practitioners only collected one PII, that is, the link to the participants’ Upwork profile. Since the study was hosted on Qualtrics, the Upwork profile was required to facilitate payment.

### Experimental Design

Assigning each participant to a different condition (noLLM, LLM) x (noDFD, DFD) x (GH, K8) would require $(2)^3$ groups. To avoid such a huge number of groups, we make use of a balanced orthogonal design, which is also known as Taguchi Design (Raghu et al. 1991). Each participant is randomly assigned to one of the four groups:

1. LLM + DFD (A) received the scenario descriptions with an accompanying data flow diagram instance and was tasked with assessing the applicability of threats using an LLM
2. noLLM + DFD (B) received the scenario descriptions with an accompanying data flow diagram instance and was tasked with self-assessing the applicability of threats
3. LLM + noDFD (C) received the scenario description without an accompanying data flow diagram instance and was tasked with assessing the applicability of threats using an LLM
4. noLLM + noDFD (D) received the scenario description without an accompanying data flow diagram instance and was tasked with self-assessing the applicability of threats

*Group formation.* In the pilot study, we configured Qualtrics to randomly assign participants to one of the four groups and balance the distribution. For the think-aloud study, the experimenters assigned the three participants to one of the groups. In the practitioner survey, similar to the pilot study, Qualtrics was also configured to assign participants to their randomised groups. However, since we could not control which participants left the study incomplete, we controlled for the distribution of the data collection campaign as it was taking place by continuously updating the group distributions in the survey software.

### Experimental Objects

To ensure the objectives of the study are met, we prepared several experimental objects.

*Scenario selection* Typically, security threats are identified against a particular system. To curate a list of threats to be used in this experiment, it was crucial to identify appropriate scenario descriptions of systems to be analysed. In addition, we considered the possibility of these systems being understood by our target population (see subsection 5.2). Following this criteria we chose two scenarios, updating a remote repository on GitHub and deploying a pod on Kubernetes. The choice to include two scenarios was to measure if the additional analysis materials are helpful in different domains. In addition, both scenarios are inspired by real open-source platforms.

*LLM selection* A myriad of Large Language Models have been developed including Google’s Gemini, OpenAI’s chatGPT, GitHub’s Copilot, and Microsoft’s Copilot among others. Since the aim of our study is not to train an LLM or compare the actual effectiveness of different LLMs, but rather to investigate their usefulness in helping human analysts in validating threats, we leverage an open-source model and opted for chatGPT-3.5 turbo model, as a first step. While the existing literature (see Section 3) has investigated the efficacy of these models in executing different tasks, a systematic comparison of their effectiveness for threat validation is an open and interesting question for future research.

*Training (4h30min)* For the pilot, the experimenters prepared a lecture on threat modeling process and landscape (2h), a training lecture with a deep dive into security threats, STRIDE, DFD, and scenarios^2^ (2h), and a walk-through presentation (30min) detailing each step of the task. The walk-through offers more understanding of what is to be expected during the actual experiment. For instance, Groups A and C’s walk-through includes an example of prompting the LLM to assess the correctness of the threat.

The training material was made available before the start of the actual task (threat validation). The process of administering the training differed slightly across all the studies (pilot, think-aloud, and practitioner survey) however, the contents of the material were the same across all three. For the pilot study, the training was delivered on two consecutive days and was also available as recordings. On the other hand, for the think-aloud and practitioners recruited from Upwork, participants were allowed to watch the training materials at their own time. In this case, there were no time limits for reviewing the training and textual materials. However, to gauge the time the participants spent on the materials, including watching the training video, they were asked to estimate and report the duration.

*Ground Truth* The ground truth has been developed systematically by the authors. First, the graphical models (DFD and sequence diagram) corresponding to each scenario description were developed by one experimenter with extensive research experience in threat analysis. Second, an initial list of bogus and realistic threats was compiled by a researcher with four years of experience in cloud security. Lastly, all the authors discussed and validated the list of threats to be used in the experiment.

- Bogus threat- We define a bogus threat as the creation of a false claim to the existence of a potential security risk.
- We present an example of a bogus threat; *Scenario: Updating a remote repository on GitHub, Threat description: An unauthenticated and nonprivileged attacker can still submit custom code into the remote repository to prepare the first step of another attack (e.g., turning off logging service or cause a Denial of Service), Assumption: The attacker can reach the remote repository (e.g. through the internet), STRIDE threat type: Elevation of privilege and Tampering, Affected DFD components: The remote code repository*

We state the justification that negates the feasibility of the threat described from being realistic *"GitHub allows owners of repositories to specify branch protection rules, which disables force pushes to the matching branches and prevents the matching branches from being deleted. When branch protection rules are implemented, an attacker cannot submit a custom code." *

*Measures of Success*

**Table 1:** Experimental variables

Name	Description	Operationalization
Independent variables (design)		
DFD	Receiving a DFD to support the threat validation	Nominal (*)
LLM	Receiving LLM API to support the threat validation	Nominal (*)
Background experience variables		
Secure design	Self-reported experience with secure design techniques	Ordinal scale ($\dagger$)
Modeling	Self-reported familiarity with design models	Ordinal scale ($\ddagger$)
Use of LLM	Self-reported frequency and the reason for using LLMs	Ordinal scale ($\dagger$)
Scenario 1	Self-reported experience with GitHub	Ordinal scale ($\dagger$)
Scenario 2	Self-reported experience with cloud deployment platforms	Ordinal scale ($\dagger$)
Dependent variables		
Actual effectiveness of threat validation	Participants actual effectiveness evaluated against a ground truth	Interval scale ($\equiv$)
Perception of additional materials	Self-reported perceived usefulness of additional analysis materials	Ordinal scale ($\dagger$)
Control measures		
Understanding	Self-reported understanding of what the task required them to do	Ordinal scale ($\ddagger$)
Time	Self-reported sufficiency of the allotted time	Ordinal scale ($\ddagger$)
Training	Self-reported sufficiency of training	Ordinal scale ($\ddagger$)
Attention checks	To account for participants understanding of the experimental objects	Interval scale ($\equiv$)
Background checks (practitioners)	To account for the professional background of industry practitioners recruited from crowd-sourcing platforms	Interval scale ($\equiv$)

(*) Qualtrics configured to automatically randomise the allocation of the independent variables. ($\dagger$) Multiple choice: For experience - attended some lecture, attended a full course, short internship, professional engagement. For frequency- never, several times a day, once a week, few times a month, once in 3 months, or "other-asked to specify". ($\ddagger$) Responses captured on a 5-point Likert scale. ($\equiv$) Responses evaluated against a predefined ground truth

Table 1 presents all the variables we consider in our experiment. This study considers two independent variables (or intervention), i.e., providing a DFD as part of the hand-out material and asking participants to perform the task of the experiment using an LLM. To achieve the aims of this study, we first measure the background experience of participants in relation to the experimental objects of the study. In this case, participants were required to self-report on their prior experience with secure design techniques, software design models, and their usage of LLMs, GitHub, and cloud deployment platforms. The responses to their background experience were captured either on a 5-point Likert scale or using predefined multiple-choice options.

Second, to answer our first research question, we analyse participants’ actual effectiveness, against the ground truth. The four possible outcomes of actual effectiveness are discussed below;

1. True Positive (TP), the number of correctly identified realistic threats according to the ground truth
2. True Negative (TN), the number of correctly identified bogus threats according to the ground truth
3. False Positive (FP), the number of bogus threats selected as being real
4. False Negative (FN), the number of real threats that were considered bogus and therefore not selected

Third, we measure the perceived usefulness of graphical models and LLMs in threat validation. To this end, participants are asked to what extent, on a 5-point Likert scale^3^, they think the additional analysis materials were useful in assisting them to correctly identify realistic threats. The responses to this question were used to answer our second research question.

Lastly, we include several control questions to account for the varying levels of comprehension of the experimental objects among the participants. These control measures consist of questions about participants’ understanding of the task, the sufficiency of time allocated to complete the task, alongside the sufficiency of the training materials. In addition, we include several attention and background checks for each of our target populations. For student participants, we measure their understanding of the experimental objects through attention checks. For industry practitioners, we use a pre-screening survey to ensure that they have the right technical background.

### Data Analysis Plan

We conducted a quantitive pilot with students, qualitative pilot (following think aloud protocol) with practitioners, and the final survey with practitioners on Upwork.

*Pilot with students and practitioner survey. * For the quantitative pilot with students, we compute descriptive statistics, conduct the analysis plan for RQ1 and RQ2 below, and analyse the responses manually. We use the same data analysis process for analysing responses of the practitioner survey.

*RQ1.* We analyse the data using the Helmert contrast, a statistical analysis used to determine the smallest shift in location of the intervention from the control treatment (Stephen and Ruberg 1989).

Helmert contrast compares each level of a categorical variable to the mean of the subsequent levels (Kaltenbach and Kaltenbach 2021). We formulate the problem for difference as;

1. noDFD&noLLM vs (noDFD&LLM union DFD&LLM)
2. noDFD&LLM vs DFD&LLM

To this end, if noDFD&LLM union DFD&LLM is greater than the control group (noDFD&noLLM), then *"some"* additional analysis material may improve the effectiveness of threat validation than having no materials at all. Similarly, if DFD&LLM is greater than noDFD&LLM, then both graphical models and LLMs may increase the actual effectiveness of threat validation as opposed to only having access to one of the additional analysis materials.

Therefore, in our study, we checked the mean of the dependent variable (actual effectiveness), for each level of additional analysis materials (i.e., Group D = no additional material, C = only LLM, and A = LLM and DFD). More specifically, the first contrast compares the mean of actual effectiveness for group D with the mean of all of the subsequent levels (Groups C and A). The second contrast compares the mean of the actual effectiveness for Group C with the mean of the subsequent levels (Group A).

When assessing the validity of security threats using LLMs (groups A and C), participants were allowed the freedom to prompt the LLM as they would in a real-world scenario. We provided them with the body of the prompt (threat description, assumption, STRIDE threat type, and affected components, see Appendix A.4) to be posted on the LLM and asked them to prompt (in their own words) the LLM for assistance. We recorded the entire LLM interaction for each security threat selected as realistic by participant.

*RQ2.* Since our data for measuring perception of usefulness is ordinal and may not be normally distributed, we use Mann Whitney U (MWU) with a level of significance equal to 0.05 ($\alpha =0.05$) to test both equivalence and difference. We formulate the problem for testing the statistical equivalence as;

$$\begin{aligned} p_{low}= & MWU (\{x - \delta | x \in A\}, B, alt = 'less') \\ p_{up}= & MWU (B,\{x + \delta | x \in A\}, alt = 'less') \end{aligned}$$

Where *A* and *B* are the vectors of the dependent variables (perceived usefulness of DFD and LLM). The $\delta$ represents the range for which we consider the means of both groups to be equivalent. The value of $\delta$ was determined before the analysis. While estimating the value of delta might seem arbitrary, similar approaches have been used in Food and Drug surveys (Meyners 2012).

*Think-aloud. * Following the best practices for conducting empirical research in software engineering (Gopstein et al. 2020; Seaman 1999; Votipka et al. 2020; Wijayarathna and Arachchilage 2019; Thompson et al. 2018), we also conduct a qualitative pilot, with a coding analysis.

The first author transcribed the recordings using Atlas.ti^4^. To maintain participants’ privacy, only the authors had access to the recordings. Thematic analysis was used to code the transcripts. A coding strategy was established before the analysis commenced. Since the think-aloud study aimed to elicit any issue that may arise before the full study was conducted, we established two high-level codes, *"Issues"* and *"What worked"*. After reading the transcripts in full, we further sub-categorised the two codes into more specific themes to capture the nuances of participant feedback. For *"Issues"*, sub-codes included *"Link accessibility"*, *"Time constraints"*, and *"Needing additional clarity of materials"*. For *"What worked"*, sub-codes included *"Appreciation of the provided materials"*, *"Clarity of provided material"*, *"Time"* and *"Use of GPT"*. In addition, to capture participants’ thought processes while validating the threats, we checked their justifications for whether a threat was marked as realistic. To this end, we added three more sub-codes to *"What worked"*, that is, *"Justification with expert knowledge"*, *"Justification with textual material"*, *"Justification with models"*.

The initial coding of the data was conducted by the first author, followed by a quality assessment performed by the second author. During the quality assessment, the second author reviewed all the coded data independently and verified the consistency of the coding process. Three disagreements (out of a total of 59 code assessments) were identified during the quality assessment process. They were subsequently discussed between both authors and resolved. The first disagreement concerned the renaming of one code. The second author agreed with the meaning of the original code *“Justification with examples”* but suggested renaming it to *“Justification with expert knowledge”* to better capture the underlying rationale. The second disagreement concerned the reference to textual materials by the participants while providing their justification for their validation of one threat. Initially, the second author was unsure what the referenced columns referred to. The first author clarified that the participant was referring to the columns in the excel file containing the threats. The columns consist of the threat description, threat assumption, STRIDE threat categories, and affected components of the system. Following this clarification, the code was confirmed as *“Justification with textual material"*. The last disagreement concerned a code of *“Justification with models"*. Initially, the second author interpreted the participants’ sentiments as referring to the limitations of DFD. Upon revisiting the recording, we confirmed that in this instance, the participant reacted to the LLM output and described a lack of data flow (i.e., information cue) in the DFD, which made them conclude about the validity of a threat.

## Pilot Study

This section presents the execution process, demographic composition, and the results of the pilot study with students.

### Execution

We present the steps taken to execute the study.

Each participant *p* joining the experiment;

1. was randomly assigned to one of the four treatment groups: A, B, C, and D, see Subsection 5.4.
2. was presented with two scenario descriptions, one on modifying and updating repositories on GitHub (GH) and the other on pod deployment on Kubernetes (K8). We configured the survey tool^5^ such that the presentation of the scenarios is randomised. That is, for two participants in the same groups, one received the Kubernetes scenario first followed by the GitHub scenario, and vice versa for the second participant.
3. was presented with a list of threats (five bogus and five realistic threats) to each scenario description.
4. was tasked with assessing the correctness of each threat and select the threats considered as realistic (likely to occur).

Each threat was accompanied by a threat description, assumptions, the associated STRIDE threat category that would be compromised if the attack occurred, and affected components. For each threat marked as realistic, participants were required to provide a short justification as to why they think it is a real threat.

### Demographics

In total, 41 participants joined the pilot study, each participant received both scenarios, so we collected in total 82 responses. Before the training, about 2/3 reported to have used GitHub in a professional capacity (13) or during an internship (12), and more than 2/3 reported having attended either a few lectures (18) or a full course on Kubernetes (10). About half reported (22) to be new to the topic of secure design and most of the participants in groups A and C reported using an LLM several times a day.

*Effect of background on actual effectiveness. * In the pilot, all participants were computer science students. We used the entry questionnaire (see Section 6.2) to ensure that their background knowledge was comparable. To this end, we do not have variability in terms of occupation, as is in the practitioner survey. We analysed the effect of their self-reported expertise in the areas relevant to this study on their effectiveness in validating threats.

Table 2 presents the results of this linear regression. We observed that having background knowledge in STRIDE ($\alpha$ = 0.013) and GitHub ($\alpha$ = 0.013) had a significant and positive effect on actual effectiveness. In contrast, participants’ familiarity with system diagrams (sequence, component, deployment, and data flow diagrams) alongside their self-reported expertise in Kubernetes did not have an effect on their actual effectiveness.

A possible explanation for the significant effect of GitHub on actual effectiveness compared to Kubernetes could be that GitHub is more commonly used among computer science students, which was the population of our pilot study. Since more students reported being novices in Kubernetes, we developed content questions for practitioner recruitment to ensure that our population in the final data collection campaign was familiar with both scenarios.

**Table 2:** Effect of self-reported expertise on actual effectiveness (TP + TN) R-Squared = 0.910

Expertise	coef	std err	t	P>|t|	[0.025	0.975]
STRIDE	3.4618	1.325	2.612	**0.013**	0.769	6.155
Sequence Diagram	2.0880	1.246	1.676	0.103	-0.444	4.620
Component Diagrams	0.4916	1.357	0.362	0.719	-2.265	3.249
Deployment Diagrams	-1.9518	1.382	-1.413	0.167	-4.760	0.856
Data Flow Diagrams	0.0910	0.939	0.097	0.923	-1.816	1.998
GitHub	1.6808	0.638	2.633	**0.013**	0.383	2.978
Kubernetes	-0.4344	0.806	-0.539	0.594	-2.073	1.204

Participants who self-reported having more background knowledge on STRIDE or GitHub performed significantly (for both, $\alpha$ = 0.013) better in validating threats. Knowledge of system components or Kubernetes had no effect on actual effectiveness

### Results

Groups that were tasked with validating security threats with the help of an LLM performed slightly better (the means for correctly identifying security threats was slightly higher in groups A ($\mu$TP$\_$A= 8.1) and C ($\mu$TP$\_$C= 9.4) compared to groups B ($\mu$TP$\_$B= 6.0) and D ($\mu$TP$\_$D= 7.4)). However, higher false positives were also reported in groups with access to an LLM (A ($\mu$FP$\_$A= 7.4) and C ($\mu$FP$\_$C= 4.7) compared to groups B ($\mu$FP$\_$B= 3.5) and D ($\mu$FP$\_$D= 3.1)). Similar observations (incorrectly assessing security information) have also been reported in prior studies on security misconceptions (Chen et al. 2023). Chen and colleagues (2023) reported that LLMs incorrectly support popular security and privacy misconceptions.

*Helmert contrast. * Table 3 contains the summary of the results from the Helmert contrast. From the results, we observe that the actual effectiveness of the group that did not receive any analysis materials to those that did (only LLM or both LLM and DFD), is not statistically different ($\alpha$= 0.505). However, a statistical difference ($\alpha$= 0.017) is observed when comparing the actual effectiveness of Group C (assessing threat validity with the assistance of LLM) to that of Group A (assessing threat validity with the assistance of LLM and in presence of a DFD). To this end, the pilot study can only partially support our alternative hypothesis ($H_{actual-eff}$).

**Table 3:** Pilot study helmert contrast with TP + TN as actual effectiveness (R-Squared= 0.190)

Levels	coef	std err	t	P>|t|	[0.025	0.975]
Intercept	13.5758	0.545	24.893	0.000	12.460	14.691
Group D vs C and A	0.4545	0.673	0.675	0.505	-0.923	1.832
Group C vs A	-0.9697	0.383	-2.535	**0.017**	-1.752	-0.187

Significant difference (α = 0.017) observed when comparing the actual effectiveness (as combined measures of TP +TN) of Group C (presence of LLM only) and A (presence of LLM and DFD)

*Observations from manual checks. * We inspected the responses manually and made some observations. However, the sample in the pilot is relatively small, and these observations need to be validated with practitioners.

Interestingly, the group with no additional material (D) reported on average the least number of FPs but still reported a relatively high number of correct threats (7.4 out of 10).

We investigated the responses of participants who received LLM advice (A, C) and observed that all participants in these groups took the first advice generated by LLM and also reported lower levels of previous knowledge about security (10 were novice to security, 9 attended some lectures, and only 2 had some hands-on experience). This could potentially also explain the higher numbers of FPs due to LLM hallucinations in those groups. In addition, when asked about the perceived usefulness of LLMs in assessing the validity of security threats after each scenario. We observed that 10/21 participants strongly agreed or agreed (points 5 and 4 on the Likert scale) with LLM’s perceived usefulness concerning the threats relating to the GitHub scenario. On the other hand, 14/21 participants strongly agreed or agreed with LLM’s perceived usefulness concerning the threats relating to the Kubernetes scenario. Thus, for security novices (e.g., practitioners in training), the use of LLM advice may still help in terms of recall (not overlooking threats) but not precision (due to a higher chance of false positives).

We also found that the presence of DFD did not make a significant difference in the collected measures of TN, FP, FN. Further, no major difference was observed for TPs when comparing the groups C and D to A and B (t-test of $\alpha$=0.05 returned a p-value of 0.048 and the Pearsons’ correlation statistic was 0.5). We conclude that no strong positive or negative correlation exists between the availability of a DFD and the actual effectiveness of correctly identifying realistic threats.

This observation indicates that, instead of the full factorial design from Table 4, it is important to observe the following progression: the differences in actual effectiveness when no material is given vs when LLM is used vs when DFD is given and LLM is used.
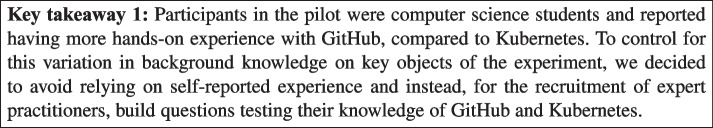

**Table 4:** Full experimental design used in the pilot and study with practitioners

	Task ($\times$ 2)		
Groups	DFD	LLM	Scenario
Group A	$\checkmark$	$\checkmark$	GH,K8
Group B	$\checkmark$	-	GH,K8
Group C	-	$\checkmark$	GH,K8
Group D	-	-	GH,K8

## Think Aloud Study

This section presents the execution process, demographic composition, and the results of the think-aloud sessions with practitioners.

### Execution

Three think-aloud sessions were conducted before the study was deployed on Upwork. The first author conducted all the think-aloud sessions. Each session took an hour to complete, and participants performed the task individually. The first author moderated the sessions by encouraging the participants to verbalise their thoughts using prompts such as "what do you think of the threat description", "why do you think this threat is real/fabricated". All sessions were audio recorded after the participants had verbalised their consent to be recorded.

All participants interested in the think-aloud study were first required to complete a pre-screening questionnaire gauging participants’ suitability for the study based on their professional background, experience, and familiarity with the experimental objects. The prescreening questionnaire was divided into several sections. First, participants answered two multiple-choice questions on general software engineering concepts, followed by professional and demographic questions. Next, participants were asked about their experience with cybersecurity risk assessment. The next section contained questions based on our study’s core concepts including their experience with Kubernetes, GitHub, and Large Language Models. Lastly, participants were asked to provide opt-in consent to participate in a think-aloud study and to provide their email addresses for contacting them in case of eligibility. We also included an example of the threat validation task they would be required to perform if invited to the think-aloud study. The pre-screening survey was deployed between June and September 2024 and received 46 entries.

During the pre-screening phase, all 46 individuals completed the survey. Before inviting participants to the study, we applied several data cleaning steps. First, we checked whether participants had consented to be invited to the think-aloud study, only 26 responded with "yes". Second, we controlled only for participants who reported having participated in security practices in their organizations and in a threat modeling session, 12 responded with "yes". Lastly, we controlled for the stage of the secure SDLC process they participate in. To this end, we only checked for participants involved in risk assessment, threat modeling and design review, or security assessment and secure configuration. This filtering step reduced the pool to 7 participants to whom we sent invitations where three accepted and took part in the study.

### Demographics

Table 5 contains an overview of the demographics of the hired participants. All three participants self-identified as male, of Asian descent, and technical cybersecurity practitioners with less than a year or up to five years of experience.

**Table 5:** Demographics of think-aloud participants

Participant	Gender	Nationality	Occupation	Years of experience
P1	Male	Malaysia	Security manager	1- 5 years
P2	Male	Pakistan	Cybersecurity consultant	< 1 year
P3	Male	Pakistan	Security manager	1- 5 years

### Results

**Table 6:** Coded participant responses (in total 59 code assessments)

Codes	P1	P2	P3	Total
Issues: Needed additional clarity for materials	5	1	4	10
Issues: Link accessibility	0	1	1	2
Issues: Time limitations	0	1	1	2
What worked: Justification with textual materials	11	7	7	25
What worked: Justification with expert knowledge	0	7	2	9
What worked: Clarity of materials	1	2	2	5
What worked: Use of GPT	0	4	0	4
What worked: Justification with models	0	1	0	1
What worked: Time	1	0	0	1

Table 6 summarises the outcome of the coding process. We made several observations from the outcomes. First, for what concerns *"Issues"*, the sub-theme *"Needing additional clarity of materials"* garnered the most codes (10), especially from P1. We observed that P1 asked confirmatory questions while reasoning, which were answered by explaining the process of the think-aloud. However, participants (P2 and P3) indicated that they would have preferred to have more information on the basics of DFDs and sequence diagrams of the Kubernetes scenario. To address the gap in the background knowledge needed to evaluate Kubernetes and GitHub threats, we developed a second, more comprehensive pre-screening survey designed to assess participants’ understanding of the two scenarios.

In addition, issues related to link accessibility (2) were mentioned by participants P2 and P3, which were fixed immediately. Time limitation (2 quotes) was also identified as an issue, with two participants indicating that the time allocated was not sufficient to complete the task comfortably. We extended the allowed time from 60 to 105 minutes for the study with practitioners.

Second, for what concerns *"What Worked"*, we found P1 found the allocated time (1 hour) to be sufficient to perform the task. In addition, all participants found the content of the materials provided to them (STRIDE training video, scenario descriptions, threat descriptions, assumptions, and the walkthrough video) relevant, useful, and prepared them well to perform the task. In addition, the clarity of materials received positive mentions (5). When justifying their evaluations, participants mainly used the information provided in the textual materials (24 quotes), followed by expert knowledge (9 quotes), and lastly, justification using DFD (1 quote).

Lastly, on the use of ChatGPT, participant *P2* mentioned that they based most of their evaluation on the information provided by the Large Language Model. However, when asked to rate the usefulness of ChatGPT in threat validation, the participant said;

*"I would say neutral, considering that it was just agreeing with everything, which I wouldn’t say is the best course."* 
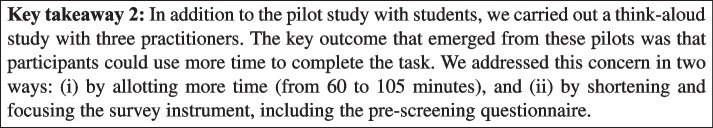


## Practitioner Survey

This section presents the execution process, demographic composition, and the results of the survey with practitioners hired from Upwork.

### Execution

The execution process defined in the pilot with students (Section 6) was closely followed. In addition, the distribution of the survey and the communication with the practitioners was conducted following the guidelines on Upwork. The group randomisation in Qualtrics had to be corrected, whenever a participant accepted, but did not complete the task.

The hiring process commenced in December 2024 and ended in April 2025. Initially, 285 participants answered the prescreening survey, but 147 responses were incomplete and thereby discarded. We then checked for participants’ actual effectiveness. Only participants who achieved a minimum of 75% of correct answers (above 15) were invited to participate in the study. Of the remaining 138 participate, 120 participants were able to correctly answer at least 15 questions. On average, participants who completed the survey and attained more than 75% correct answers took approximately 73 minutes to finish.

We sent invitations to the 120 participants, 16 declined, 26 did not respond in time, and we further removed 10 in the data cleaning process. A total of 68 participants accepted and completed the study. Removal of incomplete entries (10) affected all four groups. For Group A, one duplicate and two incomplete entries were removed. In Group B, one duplicate and three incomplete entries were discarded. In Group C, two incomplete entries were removed, while only one incomplete entry was removed from the data collected for Group D.

### Demographics

Table 7 provides an overview of participants’ distribution across all the demographic factors collected in this study including the participants’ gender, age, nationality, professional occupation, and years of experience. We observed that the majority of the participants self-identified as male (57/68) while 8 participants identified as female and 2 preferred not to disclose their gender identity.

For what concerns age, we observed that the majority (31/68) reported being between the ages of 25 and 35 while participants above 45 were the least (3/68). 17 and 16 participants reported being between 36 and 45 and under 25 years, respectively.

Table 7 contains a summary of the distribution of nationality in terms of continents. The majority of participants (36/68) reported to be from Asia, followed by Africa (15), Europe (6), North America (3), South America (3), and Oceania (1).

For what concerns their professional occupation, the study presented participants with seven predefined categories to choose from, along with an additional option that allowed them to specify their occupation if it was not included among the provided categories. From Table 7 we observed that the majority (21/68) chose to specify their occupation ("Other"). Those roles included penetration tester, network security, cybersecurity analyst, security consultant, and senior project lead among others. The second largest group in our participant pool were Security managers (18) followed by software engineers (11), DevOps engineers (7), system administrators (5), software architects (2), quality assurance/testers (2), and the least being product managers (1).

Lastly, the study also collected participants’ years of experience in five predefined categories. We observed that the majority reported having between one and five years of experience (28/68) followed by 6-10 years (20), 10-20 years (12), under 1 year (6), and the least being above 20 years (1).

Upon further investigation, we observed that, in the group with the highest number of security managers no one self-identified as female.

**Table 7:** Demographics of participants hired from Upwork

Group	Gender	No.	Age	No.	Nationality	No.	Occupation	No.	Years of exp.	No.
A	• Male	15	• Under 25	5	• Africa	2	• System Admin	1	• < 1 year	3
	• Female	2	• Between 25 - 35	8	• Antarctica		• Devops engineer	2	• Between 1-5	5
	• Non-binary	0	• Between 36 - 45	3	• Asia	11	• Software architect	1	• Between 6-10	6
	• Prefer not to say	0	• Above 45	1	• Europe	2	• Software engineer	2	• Between 10-20	3
					• North America	2	• Product manager	0	• > 20 years	0
					• Oceania (Australia)		• Q/A or Tester	0		
					• South America		• Security manager	2		
							• Other (cybersecurity)	9		
B	• Male	13	• Under 25	5	• Africa	4	• System Admin	2	• < 1 year	2
	• Female	3	• Between 25 - 35	6	• Antarctica		• Devops engineer	2	• Between 1-5	8
	• Non-binary	0	• Between 36 - 45	5	• Asia	9	• Software architect	0	• Between 6-10	4
	• Prefer not to say	1	• Above 45	1	• Europe	1	• Software engineer	6	• Between 10-20	3
					• North America	3	• Product manager	0	• > 20 years	0
					• Oceania (Australia)		• Q/A or Tester	0		
					• South America		• Security manager	4		
							• Other (cybersecurity)	3		
C	• Male	13	• Under 25	3	• Africa	3	• System Admin	1	• < 1 year	1
	• Female	3	• Between 25 - 35	9	• Antarctica		• Devops engineer	2	• Between 1-5	8
	• Non-binary	0	• Between 36 - 45	4	• Asia	9	• Software architect	1	• Between 6-10	4
	• Prefer not to say	1	• Above 45	1	• Europe	2	• Software engineer	2	• Between 10-20	3
					• North America	1	• Product manager	0	• > 20 years	1
					• Oceania (Australia)		• Q/A or Tester	2		
					• South America	2	• Security manager	3		
							• Other (cybersecurity)	6		
D	• Male	17	• Under 25	3	• Africa	6	• System Admin	1	• < 1 year	0
	• Female	0	• Between 25 - 35	9	• Antarctica		• Devops engineer	1	• Between 1-5	7
	• Non-binary	0	• Between 36 - 45	5	• Asia	8	• Software architect	0	• Between 6-10	7
	• Prefer not to say	0	• Above 45	0	• Europe	1	• Software engineer	1	• Between 10-20	3
					• North America		• Product manager	1	• > 20 years	0
					• Oceania (Australia)	1	• Q/A or Tester	0		
					• South America	1	• Security manager	10		
							• Other (cybersecurity)	3		

Majority of the participants self-identified as male with most of them being between the ages of 25 and 35. The most represented continents are Asia, Africa, and Europe. We highlight the three most represented countries in the top three continents where our participants are primarily from, Asia (India, Pakistan, Nepal), Africa (Nigeria, Kenya, Egypt), and Europe (Albania, Greece, Netherlands). Additionally, majority of the participants chose to specify their professional occupation with most of them being in the field of technical cybersecurity including penetration tester, network security, cybersecurity analyst, security consultant, and senior project lead. Lastly, most participants have between 6 and 10 years of professional experience, however, we also have some senior practitioners with more than 10 years

### Results (RQ1)

Table 8 is a summary of actual effectiveness (for comparison we also include the results from the pilot study). We observe that the means for TP for Groups A and D were similar and slightly higher ($\mu$*TP*$\_$*A and *$\mu$*TP*$\_$*D = 9.1*) compared to those of Groups B and C ($\mu$*TP*$\_$*B = 8.9 *$\mu$*TP*$\_$*C = 7.9*). On the other hand, participants of Group C ($\mu$*TN*$\_$*C = 5.4*) correctly validated more bogus threats compared to groups A, B, and D ($\mu$*TN*$\_$*A = 4.1 *$\mu$*TN*$\_$*B = 3.2 *$\mu$*TN*$\_$*D = 4.0*).

**Table 8:** Actual effectiveness of each treatment group. We report the total number of data points per group in parentheses. For instance, the number of Group A (pilot study) is 220 (11 participants each receiving 20 threats)

	TP (10 in the ground truth)					TN (10 in the ground truth)				TP+ TN			
Experiment	Group (# data points)	mean	st.dev	1st quart	3rd quart	mean	st.dev	1st quart	3rd quart	mean	st.dev	1st quart	3rd quart
Pilot (Students)	Group A (220)	8.1	1.5	7.0	9.5	3.5	2.5	2.0	4.5	11.6	2.0	10.5	12.5
	Group B (180)	6.0	1.4	5.0	6.0	6.4	2.1	5.0	8.0	12.4	2.2	11.0	14.0
	Group C (200)	9.4	0.8	9.0	10.0	5.6	2.5	4.0	7.8	15.0	2.4	14.0	16.5
	Group D (220)	7.4	2.2	6.0	9.0	6.7	2.5	5.0	9.0	14.1	4.3	10.5	18.5
Upwork (Practitioners)	Group A (340)	9.1	1.5	8.0	10.0	4.1	2.8	2.0	6.0	13.3	2.2	12.0	15.0
	Group B (340)	8.9	1.0	8.0	10.0	3.2	2.5	1.0	6.0	12.0	2.2	10.0	14.0
	Group C (340)	7.9	2.0	6.0	9.3	5.4	2.2	4.0	7.0	13.2	2.4	11.8	15.0
	Group D (340)	9.1	1.5	9.0	10.0	4.0	2.6	2.0	6.0	13.1	2.2	10.0	14.0

In the pilot study, groups assessing validity of threats using LLM (A and C) were better at identifying real threats (True Positives) while those without LLM (B and D) were better at identifying bogus threats (True Negatives). In the study with practitioners, the rate of identifying True Positives is comparable across groups A, B, and D, while Group C performed better in validating True Negatives. We obtain the mean by summing up all the correctly identified true positives divided by the number of participants

Table 9 contains the summary of the results from the Helmert contrast. The resulting coefficient of the intercept presents the overall mean of the dataset, $\mu$ $\_$Dataset = 13.1961. From the results, we observe that the actual effectiveness of the group that did not receive any additional analysis materials to those that did (only LLM or both LLM and DFD), is not statistically different ($\alpha$ = 0.386).
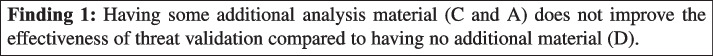


Similarly, we did not find evidence of difference ($\alpha$ = 0.827) in actual effectiveness when comparing group C (assessed threat validity with the assistance of LLM) vs group A (assessed threat validity with the assistance of LLM and in the presence of a DFD). Therefore, our data does not support our alternative hypothesis ($H_{actual-eff}$).
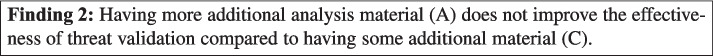


*Effect of prompting on actual effectiveness.* We analysed the chat history between the participants and the LLM to understand the impact of prompting on their actual effectiveness. Participants in groups A and C (assessing threat validity with the assistance of LLMs) were required to share the corresponding links for each interaction they had with the LLM. The total sample of links was 680, however, some of the links were inaccessible due to participants deleting their chat history. To this end, we were able to analyse 571 links. The shared links were scraped using a Python script and then manually reviewed, where we examined both the number of prompts (for each threat) and the resulting response from the LLM. To evaluate LLMs’ assessment of the validity of threats, we categorised its response based on three codes. If the LLM takes a strong stance on the validity of a threat, we used the codes *"yes"* or *"no"*. To determine this, we manually reviewed the output of the LLM history for each participant. If the LLM gives a detailed breakdown of the threat, but does not take a strong stance on whether the threat is valid, we used the code *"inconclusive"*. For quantitative analysis, we assigned each code a numeric value, *"yes"= 1*, *"no"= -1*, and *"inconclusive" = 0*.

**Table 9:** Helmert contrast

Levels	coef	std err	t	P>|t|	[0.025	0.975]
Intercept	13.1961	0.315	41.838	0.000	12.562	13.830
Group D vs C and A	0.0882	0.386	0.228	0.820	-0.688	0.865
Group C vs A	0.0490	0.223	0.220	0.827	-0.399	0.497

We did not find evidence to support our alternative hypothesis $H_{actual-eff}$. That is, the availability of additional analysis materials has no effect on actual effectiveness in threat validation

Table 10 is a summary of the resulting analysis on the average number of prompts, the assessment by participants and LLM, and the agreement rate between participants and LLM. From this analysis, we observed that on average, participants prompted the LLM once when assessing the validity of each threat. Concerning participant assessment, we observed that Group A was better at assessing real threats ($\mu$GH = 0.63 and $\mu$K8 = 0.81) while Group C was better at assessing bogus threats ($\mu$GH = 0.01 and $\mu$K8 = 0.16).
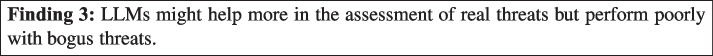


On the other hand, we observed differences in the responses of LLMs. In Group A, LLM more often correctly assessed real ($\mu$GH = 0.56 and $\mu$K8 = 0.71), while in Group C, LLM gave more inconclusive assessment for both real ($\mu$GH = 0.29) and bogus threats ($\mu$GH = 0.23) in the GitHub case scenario. Upon further investigation, we observed that when participants ask the LLM a concise question, e.g., *"Is this threat realistic and feasible in this scenario?"* the LLM provided a concrete answer as either yes, no, high, medium, or low likelihood. When the participant does not provide a concise prompt, the LLM would give a more detailed description of the threat description, assumptions, STRIDE threat type, and the affected components. This is not surprising, considering the high sensitivity of LLMs to prompts (Atzberger et al. 2025; Sclar et al. 2023).

Lastly, we observed different agreement rates between participants and the LLM. More specifically, Group A returned higher agreement rates compared to participants in Group C.
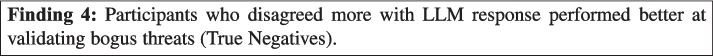


### Results (RQ2)

To answer the second research question, we reviewed participants’ responses to the perception questions.

Table 11 is a summary of participants’ perception of the usefulness of the additional analysis materials made available to them. First, the textual documentation of the threats (case study description, threat description, threat category, threat assumption, and affected components) was perceived as more useful for threat validation (point 4 and above on the Likert scale) compared to the additional analysis material (DFD or LLM). However, Group D (assessing validity of threats without LLM or DFD) perceived some textual descriptions as less useful (threat assumptions, affected components) than others.
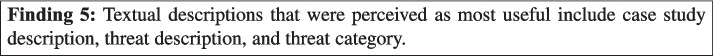


Second, for groups tasked with validating the threats with an LLM (groups A and C), group A had a slightly more positive perception than group C ($\mu$A =3.9 vs $\mu$C=3.7). On the other hand, for groups tasked with validating the threats in the presence of a DFD (groups A and B), their perceived usefulness of the diagram was the same ($\mu$=3.9).

**Table 10:** Summary of participants’ prompting of the LLM, their assessment, LLM’s assessment of threat validity, and their agreement rate(we counted agreement only in cases where LLM = 1 or 0 and the participant assessment was the same i.e 1 or 0)

Group & Scenario	Threats (# data points)	$\mu$ no. of prompts	$\mu$ assessment by participants	$\mu$ assessment by LLM	Agreement rate
Group A GitHub	Real threats (65)	1.12	0.63	0.56	0.72
Group A Kubernetes	Real threats (64)	1.09	0.81	0.71	0.76
Group A GitHub	Bogus threats (62)	1.08	0.29	0.41	0.59
Group A Kubernetes	Bogus threats (63)	1.09	0.53	0.51	0.47
Group C GitHub	Real threats (77)	1.10	0.31	0.29	0.40
Group C Kubernetes	Real threats (80)	1.06	0.59	0.48	0.45
Group C GitHub	Bogus threats (80)	1.06	0.01	0.23	0.37
Group C Kubernetes	Bogus threats (80)	1.08	0.16	0.40	0.32

**Table 11:** Result of perceived usefulness of all material provided

		Diagrams						Textual Material									
		LLM		SeqDiag		DFD		Case Desc.		Threat Desc.		Threat Category		Threat Assumption		Affected Components	
Experiment	Groups	$\mu$	$\sigma$	$\mu$	$\sigma$	$\mu$	$\sigma$	$\mu$	$\sigma$	$\mu$	$\sigma$	$\mu$	$\sigma$	$\mu$	$\sigma$	$\mu$	$\sigma$
Upwork	Group A	3.9	1.0	3.8	1.0	3.9	1.0	4.4	1.0	4.7	0.5	4.2	1.0	4.2	1.0	4.0	1.0
	Group B	-	-	3.8	1.1	3.9	1.2	4.5	0.5	4.8	0.4	4.4	0.6	4.8	0.6	4.4	0.6
	Group C	3.7	1.0	3.7	1.0	-	-	4.0	1.0	4.4	0.7	4.2	1.0	4.5	0.5	4.0	1.0
	Group D	-	-	4.0	1.0	-	-	4.1	0.7	4.4	0.7	4.0	0.7	3.6	1.0	3.6	1.0

Participants of Group A had a slightly more positive perception of LLM than Group C ($\mu$A =3.9 vs $\mu$C=3.7). On the other hand, the perceived usefulness of DFD in groups A and B was found to be the same ($\mu$=3.9)

To determine whether or not the additional analysis materials were perceived as equally useful, we performed a Mann Whitney U TOST for equivalence.

First, we checked the perceived usefulness of receiving the DFD and LLM in Group A. Table 12 presents the summary of the outcome of the test of equivalence. We observed that participants in Group A perceived the additional materials (LLM and DFD) as equally useful ($\alpha$= 0.0011).
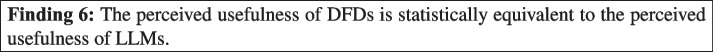


Second, we analysed the perceived usefulness of the data flow diagram in isolation (Groups A vs B). Table 13 presents the summary of the outcome of the test of equivalence. We observed a statistical equivalence ($\alpha$= 0.0019), that is, participants in groups A and B perceived the DFD as equally useful.

Lastly, we analysed the perceived usefulness of Large Language Models in isolation (Groups A vs C). The resulting analysis on Table 14 reported a statistical equivalence ($\alpha$= 0.0219), that is, participants in groups A and C perceived the LLM as equally useful. To this end, our study supports the alternative hypothesis ($H_{equiv-perc-isolation}$).
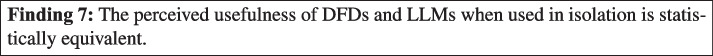


**Table 12:** Test of equivalence on perceived usefulness of LLM vs DFD in Group A

Experiment	$TOST (\delta =1)$	*MWU*	*p*
Upwork	$LLM_A$ - $\delta$ < $DFD_A$	62.5	**0.0011 ** ($p_1$)
	$DFD_A$ < $LLM_A$ + $\delta$	48.5	0.0002 $(p_2)$

Participants of Group A perceived LLM and DFD as equally useful (α = 0.0011) in performing threat validation

**Table 13:** Test of equivalence on perceived usefulness of DFD

Experiment	$TOST (\delta =1)$	*MWU*	*p*
Upwork	$DFD_A$ - $\delta$ < $DFD_B$	61.5	0.0013 ($p_1$)
	$DFD_B$ < $DFD_A$ + $\delta$	67.5	**0.0019** $(p_2)$

Participants of Groups A and B perceived the DFD as equally useful (α = 0.0019) for performing threat validation

**Table 14:** Test of equivalence on perceived usefulness of LLM

Experiment	$TOST (\delta =1)$	*MWU*	*p*
Upwork	$LLM_A$ - $\delta$ < $LLM_C$	88.0	**0.0219** ($p_1$)
	$LLM_C$ < $LLM_A$ + $\delta$	54.0	0.0006 $(p_2)$

Participants of Groups A and C perceived the LLM as equally useful (α = 0.0219) for performing threat validation

### Exit Questionnaire and Additional Controls

We analysed the responses to the exit questionnaire and performed multiple linear regressions to find potential effects of demographic factors and background on actual effectiveness and perceived effectiveness. We conclude that groups A,B,C, and D are sufficiently balanced, particularly in terms of the variables that had a significant effects (occupation and years of experience).

We also checked for first-order interactions in the regression. In the initial multiple linear regression model, we included all demographic variables (gender, age, professional occupation, years of experience, and nationality). Our participant pool is unbalanced regarding the participants’ gender, thus, we removed this variable from the model as it could not lead to meaningful conclusions. We observed that nationality had either no effect or only through an interaction. We report the interactions for completeness, but exclude nationality from Tables 15, 16, and 17 for brevity. The reference levels for the independent variables (demographics) are gender= female, age= between 25 - 35, professional occupation= DevOps engineer, years or experience= between 1-5, and nationality= Africa.

*Exit questionnaire.* We discuss the outcome of the analysis of participants’ responses to the exit questionnaire. We observed that most participants had positive answers (points 4 and 5 on the Likert scale) regarding most control questions, such as their familiarity with GitHub (64/68) and Kubernetes scenarios (54/68), and the STRIDE threat categories to understand the threat descriptions (60/68). A majority (58/68) agreed or strongly agreed (points 4 and 5 on the Likert scale) that the training video sufficiently prepared them to complete the task. Similarly, when asked if they had a clear understanding of what the task required them to do, 66 participants gave positive answers (points 4 and 5 on the Likert scale). When participants rated their perceived difficulty in identifying the correct applicable threats, only 2 selected the extreme ends of the Likert scale (points 1- very easy and 5- very hard). However, when asked how confident they were that their selections were correct, almost all of them (66/68) rated their confidence above 60%.

*Effect of demographic factors on actual effectiveness.* Table 15 contains the outcome of the linear regression. From the analysis, we observed that years of experience (between 6-10 and 10-20 years) and all professional occupations except product manager positively and significantly effected participants’ actual effectiveness. However, the lack of effect from the occupation product manager can be attributed to the unbalanced dataset, only one individual reported to be a product manager.
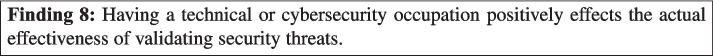


We examined the first-order interactions between years of experience and professional occupations. First, for individuals with 10-20 years of experience, significant interactions were found with other cybersecurity positions ($\alpha$ = 3.184e-04), security manager ($\alpha$ = 4.259e-04), and software engineer ($\alpha$ = 2.006e-02). Second, for those with 6-10 years of experience, significant interactions emerged with other cybersecurity positions ($\alpha$ = 9.612e-06), quality assurance/tester ($\alpha$ = 1.784e-02), security manager ($\alpha$ = 7.161e-05), system administrator ($\alpha$ = 4.285e-03), and software architect ($\alpha$ = 1.561e-02).
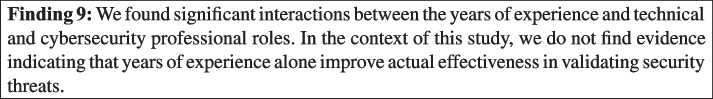


*Effect of demographic factors on perception of additional analysis materials.* We classified the additional materials into two categories, models and textual. The large language model used and the data flow diagram were classified as models. On the other hand, the case description, threat descriptions, threat categories, threat assumptions, and the affected components were classified as textual. To perform the linear regression, we first calculated the mean perception scores for each participant’s responses to their perception of each variable for each category of model or textual materials. The participants’ demographic factors were then analysed against the resulting aggregated means.

**Table 15:** Effect of demographic factors on actual effectiveness (TP + TN) R-Squared = 0.922. The reference levels for the independent variables (demographics) are age= between 25 - 35, professional occupation= DevOps engineer, and years of experience= between 1-5

Demographics	coef	std err	t	P>|t|	[0.025	0.975]
Age$\_$36 - 45	-0.0562	1.500	-0.037	0.970	-3.064	2.951
Age$\_$Above 45	-0.4496	3.458	-0.130	0.897	-7.383	6.484
Age$\_$Under 25	2.8269	1.677	1.685	0.098	-0.536	6.190
Occupation$\_$Other cybersecurity roles	11.5031	1.147	10.028	**6.189e-14**	9.203	13.803
Occupation$\_$Product Manager	8.0315	4.316	1.861	0.068	-0.622	16.685
Occupation$\_$Quality Assurance/Tester	8.6023	3.143	2.737	**8.379e-03**	2.301	14.904
Occupation$\_$Security Manager	10.1112	1.237	8.177	**5.111e-11**	7.632	12.590
Occupation$\_$Software Architect	7.6713	3.302	2.323	**2.394e-02**	1.052	14.291
Occupation$\_$Software Engineer	8.1714	1.881	4.345	**6.187e-05**	4.401	11.942
Occupation$\_$System Administrator	9.8535	2.069	4.763	**1.472e-05**	5.706	14.001
Exp$\_$10 - 20 years	4.3385	1.873	2.316	**0.024**	0.584	8.093
Exp$\_$6 - 10 years	3.9685	1.346	2.949	**0.005**	1.270	6.667
Exp$\_$Less than a year	3.9181	2.099	1.867	0.067	-0.290	8.126
Exp$\_$More than 20 years	4.9466	5.487	0.901	0.371	-6.055	15.948

All professional occupations (except product manager) and some years of experience (between 6-10 years and 10-20 years) positively and significantly affected participants actual effectiveness

Table 16 presents the outcome of the linear regression on the effect of demographic factors on the perception of models (LLM, DFD, Sequence diagram). We observed that, certain professional occupations (other technical cybersecurity positions, quality assurance/tester, security manager, software engineer, and system administrator), and Nationality (participants from Asia $\alpha$= 0.008 and North America $\alpha$= 0.006) positively and significantly effected participants perception of the usefulness of models to perform the task.
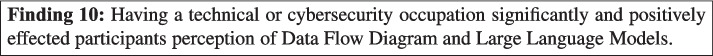


We examined the first-order interaction between Nationality and professional occupations. For Asian practitioners, a significant interaction emerged with other cybersecurity positions ($\alpha$ = 8.388e-05), security manager ($\alpha$= 1.647e-02), and software engineer ($\alpha$ = 1.230e-03). On the other hand, for North American practitioners, a significant interaction was observed with other cybersecurity positions ($\alpha$ = 7.537e-03) and software engineer ($\alpha$ = 2.025e-02) only.
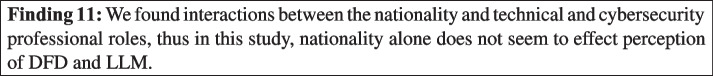

**Table 16:** Effect of demographic factors on perception of models (LLM, DFD, Sequence diagram) R-Squared = 0.910. The reference levels for the independent variables (demographics) are age= between 25 - 35, occupation= DevOps engineer, and years of experience= between 1-5

Demographics	coef	std err	t	P>|t|	[0.025	0.975]
Age$\_$36 - 45	-0.0017	0.596	-0.003	0.998	-1.200	1.196
Age$\_$Above 45	-0.9042	1.278	-0.708	0.482	-3.470	1.662
Age$\_$Under 25	0.2876	0.643	0.447	0.656	-1.004	1.579
Occupation$\_$Other cybersecurity roles	2.5801	0.423	6.093	**1.571e-07**	1.730	3.431
Occupation$\_$Product Manager	2.3765	1.463	1.624	0.111	-0.562	5.315
Occupation$\_$Quality Assurance/Tester	2.1382	1.076	1.988	**5.230e-02**	-0.022	4.299
Occupation$\_$Security Manager	2.8052	0.440	6.378	**5.634e-08**	1.922	n3.689
Occupation$\_$Software Architect	1.9906	1.254	1.588	0.119	-0.528	4.509
Occupation$\_$Software Engineer	2.0747	0.646	3.212	**2.306e-03**	0.777	3.372
Occupation$\_$System Administrator	2.2392	0.719	3.115	**3.044e-03**	0.795	3.683
Exp$\_$10 - 20 years	0.2983	0.676	0.441	0.661	-1.060	1.657
Exp$\_$6 - 10 years	0.3112	0.529	0.588	0.559	-0.752	1.374
Exp$\_$Less than a year	0.6536	0.735	0.890	0.378	-0.822	2.130
Exp$\_$More than 20 years (Please specify)	0.9121	0.969	0.942	0.351	-1.033	2.857

All professional occupations (except product manager and software architect) positively and significantly effected participants’ perception of the usefulness of models

Table 17 presents the outcome of the linear regression on the effect of demographic factors on the perception of textual materials. We observe that several roles (quality assurance, security manager, software architect, software engineering, system administrator, and other technical cybersecurity occupations) have a positive and significant effect on the perception of the textual materials.
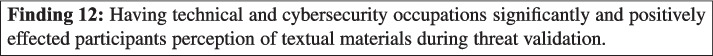


In addition, only participants with 6-10 years of professional experience perceived the available textual materials as more useful, albeit the differences are small. We examined whether an interaction exists between the years of experience and the professional occupations. The results of the first-order interaction did not result in any statistical significance.

**Table 17:** Effect of demographic factors on perception of textual materials (case description, threat descriptions, threat categories, threat assumptions, and the affected components) R-Squared = 0.916. The reference levels for the independent variables (demographics) are age= between 25 - 35, occupation= DevOps engineer, and years of experience= between 1-5

Demographics	coef	std err	t	P>|t|	[0.025	0.975]
Age$\_$36 - 45	0.4911	0.505	0.972	0.335	-0.522	1.504
Age$\_$Above 45	0.1557	1.165	0.134	0.894	-2.179	2.490
Age$\_$Under 25	0.7782	0.565	1.378	0.174	-0.354	1.911
Occupation$\_$Other cybersecurity roles	3.4302	0.386	8.881	**3.840e-12**	2.656	4.205
Occupation$\_$Product Manager	2.1716	1.453	1.494	0.141	-0.742	5.085
Occupation$\_$Quality Assurance/Tester	3.8967	1.058	3.682	**5.365e-04**	1.775	6.019
Occupation$\_$Security Manager	3.3725	0.416	8.100	**6.812e-11**	2.538	4.207
Occupation$\_$Software Architect	2.2887	1.112	2.059	**4.436e-02**	0.060	4.518
Occupation$\_$Software Engineer	3.3179	0.633	5.239	**2.731e-06**	2.048	4.588
Occupation$\_$System Administrator	3.3873	0.697	4.863	**1.039e-05**	1.991	4.784
Exp$\_$10 - 20 years	0.9718	0.631	1.541	0.129	-0.293	2.236
Exp$\_$6 - 10 years	1.0284	0.453	2.269	**0.027**	0.120	1.937
Exp$\_$Less than a year	1.1247	0.707	1.591	0.117	-0.292	2.542
Exp$\_$More than 20 years (Please specify)	0.8141	1.848	0.441	0.661	-2.891	4.519

All professional positions (except product manager), and 6-10 years of experience positively and significantly effect participants’ perception of textual materials

*Effect of prompt on agreement rate.* The participants in this study were provided with an example prompt to use, but to provide realistic usage, we allowed to freely interact with the LLM. The variability in this interaction could have effected the quality of the LLM response. From our analysis of the prompt history, we could conclude that while some participants prompted twice (with some differences in the prompt length), their prompts did not significantly differ in terms of prompt content (e.g., none of them tried few-shot learning, or other prompt engineering techniques). We investigated the relationship between the length of the participants’ prompt to their agreement with the judgment of the LLM on the validity of threats. To this end, we computed a Pearson correlation between the average number of words used in a prompt and the agreement rate aggregated by threat group (actual and fabricated threats).

Table 18 is a summary of the mean agreement rate and the average number of words used in prompts. From the results, we observed that, for the GitHub scenario, Group A generally had a higher agreement rate and used slightly more words compared to Group C. Furthermore, participants seemed to agree more with the judgment of LLM on the validity of threats for actual threats (threat group 1-5) for both groups. The Pearson correlation for this scenario is 0.72 ($r = 0.72$), which indicates a strong positive correlation, however, not significant (p-value = 0.281).

The results of the Kubernetes scenario are such that participants in Group C used slightly more words in their prompts, yet, on average, agreed less with the judgment of LLM on threat validity. On the other hand, Group A had a slightly higher agreement rate, especially for actual threats (threat group 1-5). The Pearson correlation for this scenario is -0.15 ($r = -0.15$), which indicates a weak negative correlation, however, not significant (p-value = 0.851).

The differences in agreement across threat groups 1-5 and 6-10 across both scenarios and treatment groups additionally corroborate the observation reported in Finding 3.

**Table 18:** Length of prompt and agreement rate (Threat group 1-5= Actual threats, Threat group 6-10 = Fabricated threats)

Scenario	Group	Threat Group	Agreement	Average words
GitHub	A	1-5	0.72	79.69
	A	6-10	0.59	84.29
	C	1-5	0.40	72.83
	C	6-10	0.37	75.60
Kubernetes	A	1-5	0.76	92.65
	A	6-10	0.47	90.50
	C	1-5	0.45	95.37
	C	6-10	0.32	93.10

In the GitHub scenario, there was a strong positive correlation ($r= 0.72$) between the length of the prompt to participants’ agreement with the judgement of LLM on threat validity, however, not significant ($p= 0.281$). On the other hand, in the Kubernetes scenario, a weak negative correlation ($r= -0.15$) between the length of prompt to participants’ agreement with LLM advice was observed, albeit not significant ($p= 0.851$)


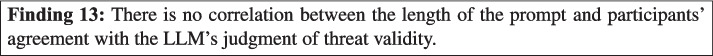


## Discussion

We provide answers to the research questions, contextualize the main results with related work, and conclude by discussing the generalizability of the results beyond security threat validation.

### Answers to Research Questions

In the pilot with students, we found that using LLM only (Group C) improves threat validation, and that the student participants tend to agree with the LLM response. A possible explanation for the higher agreement rate could be related to trust. But, the LLM may provide an illusion of competence by using persuasive technology or overconfident actual effectiveness (Wester et al. 2024; Yin et al. 2019). Indeed, this finding was not confirmed in our expert population. Participants in Group C performed equally well, but the practitioners agreement rate with LLM response is fairly low. This was confirmed during the think-aloud study, where participants mainly relied on their own expert knowledge to justify their assessment.

On the other hand, we observed that the presence of a DFD only made a small difference in the actual effectiveness (as True Positives) of students. For expert practitioners, we did not observe any significant difference in the actual effectiveness when assessing threat validity in the presence of a DFD, see Table 23 in Appendix A.2. Related literature has also found that having only a graphical representation does not increase productivity. For instance, based on the findings by Van Landuyt and Joosen (2022), the authors advice against the exclusive use of Data Flow Diagrams during threat analysis but recommend integrating other system models. Similarly, Labunets et al. (2017) found that the presence of a graphical representation alone does not necessarily improve security requirements analysis.

*RQ1: What is the actual usefulness of having additional material like DFD or LLMs during threat validation?* 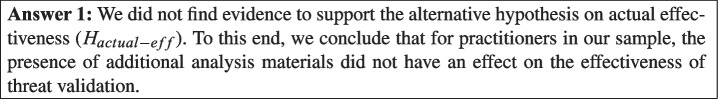


In other words, there is no need to use the DFD or an LLM to validate threats.

*Contextualization with related work.* Previous work (Karpati et al. 2014; Andreas et al. 2009) found that attack trees (a type of model) resulted in the identification of a higher number of threats compared to misuse cases (structured text). On the other hand, Labunets et al. (2017) studied the difference in effectiveness of graphical and tabular techniques for security risk analysis and found that they are statistically equivalent in terms of effectiveness. Similar to Labunets (2017), we found no difference (in terms of effectiveness for threat validation) when using additional modeling (DFD) or textual (advice from LLM) material.

Community knowledge in the form of security patterns or security catalogs are used for security risk analysis (Souag et al. 2015) and is known to improve organizational effectiveness and competitiveness (Lynne and Markus 2001). We contextualize our findings in the security knowledge reuse space, as by using an LLM we explored one querying mechanism of public security sources (such as CWE MITRE 2020, CAWE Joanna et al. 2017,CAPEC Barnum 2008). Allodi et al. (2020) reports that knowledge and skills are important factors to consider in the accuracy of software vulnerability assessments. More specifically, they observed that for specific CVSS metrics (i.e., user interaction), professionals were more accurate in their assessment due to their domain-specific knowledge. Labunets et al. (2023) also find that experts rely on their own knowledge as a source of information and navigate the catalogues to check their intuition. Our results confirm these findings.

From the additional analysis of our control variables in Section 8.5, we found that the most consistent predictor for higher actual effectiveness was the type of occupation. Specifically, a participant reporting a security (security manager, other cybersecurity roles such as penetration tester, network security engineer) or technical role (tester, developer, system administrator) in organizations had a positive effect on how well they did in validating the threats. This is in line with the past literature on threat modeling (Soares Cruzes et al. 2018; Shostack 2014; Tuma et al. 2018) and experience reports from practice (Dhillon 2011).

*RQ2: What is the perceived usefulness of the additional material during threat validation?*

Expert practitioners perceived textual materials (case study description, threat description, and threat category ) as more useful on average compared to the additional material (DFD or LLM). We found significant equivalence when comparing the perceived usefulness of the Data Flow Diagrams and Large Language Models, both in isolation and when used together. Hence, both alternative hypotheses ($H_{equiv-perc-both}$, $H_{equiv-perc-isolation}$) for the perceived usefulness of additional analysis materials was upheld.
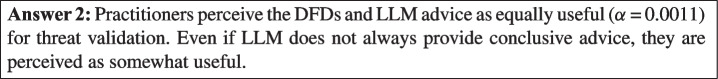


One explanation could be that prompting a large language model can trigger critical thinking. Future research is needed to validate this further.

*Contextualization with related work. * Previous research (Van Landuyt and Joosen 2022; Mbaka et al. 2025) observed that textual descriptions are an integral part for assessing security threats. For instance, the study by Van Landuyt and Joosen (2022) focuses on the application of threat assumptions in practice. The study posits that the use of assumptions is essential since security decisions are often based on the credibility of the information conveyed by the assumptions (Van Landuyt and Joosen 2022). Our results confirm these findings.

In addition, several studies have investigated the perceived usefulness of graphical versus textual representation on various security domains such as requirements comprehension (Sharafi et al. 2013) and risk assessment (Labunets et al. 2017). Similar to the conclusions made in our study, both (Sharafi et al. 2013) and (Labunets et al. 2017) found evidence of statistical equivalence in the perception of graphical and textual representations.

Chen et al. (2023) found that LLMs can be noncommital (do not take a strong stance) when asked to judge security and privacy misconceptions. Similarly, we observed that when the LLM was noncommital, the participant had to rely on their own understanding of the provided materials or their expertise to validate threats.

But, we have not found our participants to have more negative perception of chatGPT compared to other material. Even when they disagreed with the recommendation, they still perceived it as useful, which is interesting, as it might indicate that practitioners are using it as a tool to critically think about threats.

### Generalization

The findings derived from investigating RQ1 and RQ2 are limited to the observed population (majority male, 25-30 years old, with a security role) and the task of validating the already identified security threats as true positives and true negatives. The results may generalize to other security risk analysis tasks, particularly when information is being validated. For instance, validating vulnerability risk assessments (Holm and Afridi 2015; Zhang et al. 2023), or validating threat intelligence where reliance on human competence and intuition is still prevalent in practice (Geras and Schreck 2024; Shu et al. 2018). Our work only measures true positives and true negatives, so our findings may not generalize to scenarios where the analysts need to filter for false positives. For example, our study does not explore whether additional material helps the analysts sieving through security alerts to check if they are false alerts (Tariq et al. 2025). Similarly, these findings may not generalize to threat identification, where the goal is minimizing false negatives to avoid overlooking important security threats (Soares Cruzes et al. 2018; Bernsmed and Jaatun 2019; Labunets et al. 2023). Finally, our findings on the perceived usefulness of additional material might generalize to other validation tasks in security risk analysis (Labunets et al. 2023).

## Threats to Validity

This section discusses the planned mitigations to potential internal and external threats to validity.

*Internal validity. * To ensure that the complexity of the two scenarios is comparable, the DFDs have the same graph topology. Namely, both DFDs contain 3 data store nodes, 1 external entity node, 6 process nodes, and 16 data flows connecting the same type of nodes. In addition, we ensured the complexity of the task were comparable across treatment groups. We acknowledge that other factors, such as participants’ technical and cognitive skills may influence their understanding of the task and ultimately affect its complexity. To mitigate this risk, we used the entry questionnaire in the pilot and pre-screening questionnaire for Upwork to measure participants’ familiarity with the experimental objects.

We also consider the threat of introducing experimenter bias in the ground truth. To mitigate this threat, we built the material carefully involving four researchers. The DFD and list of security threats were built by two experimenters, one with more than 8 years of experience in threat analysis, and one with more than 4 years of experience with Kubernetes and cloud security. The ground truth was verified with two other members of the group (junior, and senior with $10+$ years of experience in controlled experimentation).

From the control measures, 29/41 participants from the pilot study and 66/68 practitioners from Upwork reported either strongly agreeing or agreeing (points 5 and 4 on the Likert scale) to have a good understanding of what the task required them to do. In addition, 30/41 students from the pilot study either strongly agreed or agreed that the time allowed for the experiment was sufficient. On the other hand, practitioners from Upwork reported on average to spend between one to two hours to read the materials before performing the task. To this end, we conclude that the training material and the time allowed to finish the task were sufficient for our target population.

Another threat to validity pertains to how training was delivered across the three studies. In the pilot, the participants received the training in person, whereas for the think-aloud and Upwork study, practitioners received online links to the training materials and were asked to review them before starting the task of threat validation. We were unable to fully validate whether or not practitioners watched the materials to their entirety. To mitigate this threat, we implemented a series of control measures. A pre-screening questionnaire was administered for both participants from the think-aloud and Upwork studies, containing technical questions on the key concepts of the study. Second, during the think-aloud study, the experimenters directly interacted with the participants and observed that they had an understanding of the study’s core concepts. Lastly, we only invited participants who scored 75% and above in the pre-screening survey.

During data collection, the first author checked participant submissions and confirmed that all links were accessible before ending their contractual agreement on Upwork. During data analysis we observed that some links (109 out of 680) of the shared LLM chat history were unavailable. A possible explanation for the inaccessible links would be that the participants deleted their chat history. The responses of those participants were excluded in the analysis of LLM prompting.

Since participants were allowed the freedom to formulate their own prompts, having only been provided with the body of the prompt, we could not control for the outcomes of the LLM. To this end, it is possible that differences in the length of prompts, choice of words used, or depth of additional details provided could influence the LLM’s output. As a result, participants’ prompting strategies may have also influenced their agreement rate with the LLM (as discussed in Subsection 8.3). To mitigate this threat, we developed a ground truth that allowed us to objectively evaluate participants’ submissions regardless of variations in their prompting. As a control, we also analysed the effect of prompts on participants’ agreement with the judgement of LLM on the validity of threats. We found no correlation between the length of the prompt and participants’ agreement rate.

*External validity. * We are aware of the challenges in recruiting participants from crowd-sourcing platforms (Reid et al. 2022; Ebert et al. 2022), such as, participants self-reporting on their levels of expertise or background knowledge without having to provide evidence. To mitigate this challenge, we pre-screened the participants as recommended by Alami and colleagues (Alami et al. 2024). The pre-screening layer included questions testing the knowledge of participants on the scenarios used in the task.

We consider the threat of generalisability of our findings to real-world scenarios. To partially mitigate this risk, we use application of K8 pod deployment and Github remote repository update, two realistic and common tasks in software development, and allow participants to use LLMs in a controlled but not limiting way.

Since students participating in empirical studies have been reported to have a good understanding of industry-level requirements (Svahnberg et al. 2008), we decided to invite students in the first pilot study, but conducted the final study with practitioners.

Our participation pool was predominantly male, a challenge that may introduce demographic bias into our findings. The issue of unbalanced gender populations is already well understood in STEM disciplines (Rodríguez-Pérez et al. 2021), and computer science is no exception. To this end, we decided to remove this variable when analysing the effect of demographic factors on actual and perceived effectiveness. As such, our findings may not fully represent the effect of demographic factors on threat validation. This limitation highlights the need for future replication with a more balanced participant pool.

## Conclusion and Future Work

Security analysts validate the identified security threats to assert that they have done a "complete job" and have not overlooked any important security threat. The aim of this work was to find empirical evidence of what material must the analysts use to effectively validate the identified security threats.

We conducted a controlled experiment measuring the actual and perceived effectiveness of additional analysis materials (DFD and LLM) when validating security threats. First, we carried out a pilot study with 41 Msc computer science students and a think-aloud study with three practitioners. To conduct the study, we made use of a balanced orthogonal experimental design where each participant was randomly assigned to one of four groups (A, B, C, or D). All participants received the same experimental material (two case study descriptions, list of threats corresponding to each case study, sequence diagram, training on STRIDE, DFD, and additional material on threat analysis). In addition, depending on the randomised group, participants received additional analysis material (A- DFD and LLM, B- DFD only, and C- LLM only), group D (intervention group) did not receive any additional materials.

The result of our experiment is that having additional analysis material does not improve the actual effectiveness of threat validation for students or practitioners. In other words, less is more. Interestingly, the DFDs were perceived as equally useful compared to LLM advice for threat validation.

These findings were derived within the population represented in our data set (majority male, 25-30 years old, with a security role), but might generalize to other security risk analysis tasks, particularly where information is being validated. Replications with a more balanced population (in terms of gender and occupation) are required to better understand the effect of these variables on threat validation. Moreover, since we found evidence of the perceived usefulness of additional analysis materials in threat validation, an interesting future direction would be to investigate to what extent security analysts have adapted using AI as assistants in the brainstorming and validation of security threats. In addition, it would be interesting to measure the existence of bias (if any) towards adaptation of AI (appreciation vs aversion) and the effect either has on the actual effectiveness of threat analysis and risk assessment when using AI assistants.

## Data Availability

For replicability, we have provided a replication package (Bahati Mbaka andTuma 2025) where we include the: (i) training materials in PDF form, (ii) pre-screening questions for recruiting industry practitioners, (iii) the survey instrument (including embedded links to the videos), (iv) the scenario documentations, (v) coding analysis from the think aloud study, and (vi) python scripts for the data analysis. The complete responses of all participants are available upon request.

## A: Appendix

This section presents additional analysis of actual effectiveness.

### A.1 Helmert Contrast on Pilot Study

We performed the Helmert contrast on the actual effectiveness of participants of the pilot study. Tables 19, 20, and 21 outline the results of this statistical test. We checked for statistical significance systematically. First, we performed the Helmert contrast across three groups (A, C, and D), following the evidence supporting the lack of strong positive correlation between the availability of a DFD and actual effectiveness of correctly identify realistic threats outlined in Section 6. Second, we performed the Helmert contrast across all four groups, see Table 21.

From the first Helmert contrast (Tables 19 and 20 we observed a significant difference between the actual effectiveness of Group D vs C and A (p-value = 0.007) when measured as TP only. On the other hand, we observed a significant difference between the actual effectiveness of Group C and that of Group A when measured as TN (p-value = 0.009) Table 20.

**Table 19:** Helmert contrast with TP as actual effectiveness (R-Squared= 0.226)

*Levels*	coef	std err	t	P>|t|	[0.025	0.975]
Intercept	8.284	0.286	28.949	0.000	7.700	8.870
Group D vs C and A	1.018	0.353	2.882	**0.007**	0.296	1.741
Group C vs A	-0.097	0.201	-0.483	0.633	-0.508	0.314

A significant difference was observed when comparing the actual effectiveness (as True Positives α = 0.007) of Group D (without any additional analysis materials) to Groups C (presence of LLM only) and A (presence of LLM and DFD).

**Table 20:** Helmert contrast with TN as actual effectiveness (R-Squared= 0.236)

Levels	coef	std err	t	P>|t|	[0.025	0.975]
Intercept	5.290	0.447	11.828	0.000	4.376	6.206
Group D vs C and A	-0.563	0.552	-1.021	0.316	-1.693	0.566
Group C vs A	-0.872	0.314	-2.782	**0.009**	-1.514	-0.231

The actual effectiveness of Group C was found to be significantly different (α = 0.009) from Group A when comparing participants’ True Negatives

In the second analysis, we observed two significant differences when actual effectiveness was measured as TP, first when comparing Group D vs the means of Groups C, B, and A (p-value = 0.005), and second when comparing the actual effectiveness of Group C to the average actual effectiveness of Groups B and A (p-value = 0.001). In addition, the actual effectiveness of Group B was found to be significantly different from that of Group A when measured as True Negatives (p-value = 0.003). Lastly, when actual effectiveness is measured as TP+TN, the actual effectiveness of Group B was found to be significantly different from that of Group A (p-value = 0.038).

**Table 21:** Helmert contrast on pilot study with 4 levels on actual effectiveness as either TP, TN, and TP+TN

Levels	coef	std err	t	P>|t|	[0.025	0.975]
(a) Helmert contrast with TP as actual effectiveness (R-Squared=0.386)						
Intercept	7.713	0.247	31.240	0.000	7.213	8.214
Group D vs C, B, A	1.018	0.344	2.958	**0.005**	0.321	1.716
Group C vs B, A	-0.793	0.209	-3.793	**0.001**	-1.218	-0.370
Group B vs A	0.125	0.139	0.905	0.371	-0.156	0.407
(b) Helmert contrast with TN as actual effectiveness (R-Squared= 0.233)						
Intercept	5.579	0.382	14.616	0.000	4.806	6.353
Group D vs C, B, A	-0.563	0.532	-1.059	0.296	-1.642	0.515
Group C vs B, A	0.093	0.324	0.289	0.774	-0.562	0.749
Group B vs A	-0.677	0.215	-3.156	**0.003**	-1.113	-0.243
(c) Helmert contrast with TP+TN as actual effectiveness (R-Squared= 0.188)						
Intercept	13.292	0.456	29.133	0.000	12.368	14.217
Group D vs C, B, A	0.454	0.636	0.715	0.479	-0.834	1.743
Group C vs B, A	-0.700	0.387	-1.811	0.078	-1.484	0.083
Group B vs A	-0.552	0.257	-2.150	**0.038**	-1.073	-0.032

A significant difference was observed when *i)* comparing the actual effectiveness (as TP α = 0.005) of Group D vs A, B, and A *ii)* Group C vs B and A *iii)* comparing the actual effectiveness (as TN α = 0.003) of Group B vs A and *iv)* comparing the actual effectiveness (TP α = 0.001) (as TP + TN α = 0.038) of Group B vs A

### A.2 Additional Analysis on Upwork Data

Table 22 presents the results of the Helmert contrast performed on the individual measures of actual effectiveness, True Positives (Table 22a) and True Negatives (Table 22b). Similar to the observations made about the combined measures of TP + TN outlined in Subsection 8.3, no significant difference were observed between the group that did not receive additional analysis materials (Group D) and those that received an LLM (Group C) or both LLM and DFD (Group A). In addition, no significant difference was observed between the groups that received additional analysis materials (Groups C vs A).

**Table 22:** Helmert contrast with 3 levels on actual effectiveness as either TP or TN

*Levels*	coef	std err	t	P>|t|	[0.025	0.975]
(a) Helmert contrast with TP as actual effectiveness (R-Squared=0.091)						
Intercept	8.725	0.235	37.197	0.000	8.254	9.197
Group D vs C and A	-0.529	0.287	-1.843	0.072	-1.107	0.048
Group C vs A	0.196	0.166	1.182	0.243	-0.137	0.530
(b) Helmert contrast with TN as actual effectiveness (R-Squared=0.045)						
Intercept	4.470	0.361	12.374	0.000	3.744	5.197
Group D vs C and A	0.617	0.442	1.396	0.169	-0.272	1.507
Group C vs A	-0.147	0.255	-0.576	0.568	-0.661	0.367

No significant difference found when comparing the actual effectiveness (TP or TN) of the group with no additional analysis materials (Group D) and those that received an LLM (Group C) or both LLM and DFD (Group A) or between the groups with some additional analysis materials (Groups C vs A)

Table 23 presents a summary of the resulting outcome of Helmert contrast across all groups (A, B, C, and D) and actual effectiveness as either True Positive (Table 23a), True Negative (Table 23b) and the summation of TP and TN (Table 23c). From the results, we observe a significant difference only when comparing the actual effectiveness (TP and TN in isolation) of Group C (assessing validity of threats with LLM only) against that of Groups B and A (*p*-value$_{TP}$ = 0.036, *p*-value$_{TN}$ = 0.034). However, no significant difference was observed across all groups when the measures of actual effectiveness were combined (TP + TN), sub-table Table 23c.

**Table 23:** Helmert contrast with 4 levels on actual effectiveness as either TP, TN, and TP+TN

Levels	coef	std err	t	P>|t|	[0.025	0.975]
(a) Helmert contrast with TP as actual effectiveness (R-Squared=0.085)						
Intercept	8.764	0.185	47.453	0.000	8.396	9.134
Group D vs C, B, A	-0.088	0.261	-0.338	0.737	-0.610	0.434
Group C vs B, A	-0.323	0.151	-2.145	**0.036**	-0.625	-0.022
Group B vs A	0.117	0.107	1.103	0.274	-0.095	0.331
(b) Helmert contrast with TN as actual effectiveness (R-Squared= 0.080)						
Intercept	4.147	0.310	13.381	0.000	3.528	4.766
Group D vs C, B, A	-0.411	0.438	-0.939	0.351	-1.287	0.464
Group C vs B, A	0.549	0.253	2.170	**0.034**	0.044	1.055
Group B vs A	0.009	0.179	0.055	0.956	-0.348	0.367
(c) Helmert contrast with TP+TN as actual effectiveness (R-Squared= 0.050)						
Intercept	12.911	0.273	47.287	0.000	12.366	13.457
Group D vs C, B, A	0.088	0.386	0.228	0.820	-0.683	0.860
Group C vs B, A	-0.362	0.223	-1.627	0.109	-0.808	0.083
Group B vs A	0.127	0.158	0.808	0.422	-0.187	0.442

Significance difference was only observed when comparing the actual effectiveness (TP α = 0.036 and TN α = 0.034 in isolation) of Group C (assessing threat validity with the assistance of LLM) to that of Group B (assessing threat validity in presence of a DFD) and Group A (assessing threat validity with the assistance of LLM and in presence of a DFD)

### A.3 Information Cues and Technical Knowledge for Validating Threats

To assist in the validation of security threats, several experimental objects, outlined in Subsection 5.5 were made available to the participants. Using these experimental objects, we extracted the information cues that we deemed necessary to effectively complete the task. Tables 24 and 25 outline the pieces of information that were deemed necessary in aiding in the evaluation of the validity of threats. The tables correspond to each scenario, GitHub (Table 24) and Kubernetes (Table 25).

For each threat, we first present the justification for our ground truth which led to their classification as either *actual* or *fabricated*. Second, if the scenario description or the assumptions contains information that would guide the participant in making a judgment, then we indicate it under the textual hint column. Lastly, we consider the study’s intervention, the data flow diagram. To this end, if a logical information flow is presented in the graphical diagram, we indicate it under the additional hint (DFD) column.

Additionally, Tables 26 and 27 present the technical knowledge deemed necessarily to aid in the judgment of the validity of each threat.

**Table 24:** Information needed to judge Validity of threats- GitHub scenario (For the column Actual, 1= actual threat, 0= fabricated threat)

ID	Actual	Justification	Textual hint	Additional hint (DFD)
1	1	Using simple alert-style email notifications, attackers are able to steal credentials to gain access to development code, intellectual property, and project details	Assumptions confirms a successful social engineering attack	Absence of a multi-factor authentication process
2	1	Git’s configuration files are plain-text, when leaked an attacker can view the personal information of the repository owner, leading to information disclosure attack. In addition, the attacker can alter the default behaviour set by the owner by manually editing these values	The YAML configuration files are automatically executed	Presence of configuration file data store
3	1	An attacker can exploit vulnerabilities (e.g., lack of authentication) in the git protocol to send multiple requests that overload or crash the server causing a DoS attack	Assumption confirms attacker’s ability to submit malicious code	Absence of authentication process
4	1	An attacker can get server and .../path/to/repo from git remote (only if the remote repository is of the ssh type). This permits the execution of server-side Git commands implementing the pull/push functionality	Assumptions confirm that server has pulled and is running malicious code	Presence of run new push data flow
5	1	With root access, the attacker is able to perform any actions on GitHub, such as creating a pull/push request in a repository	Assumption that the attacker can submit code remotely	Presence of a commit and push data flow
6	0	It is possible for an attacker to submit a fake push request to other remote code repositories if they have access to the necessary credentials or exploit a vulnerability in the system. This could potentially cause a Denial of Service attack. But the assumptions clearly states that permission management is properly implemented	Confirmation of properly implemented permission management	Presence of a protocol process which executes push commands from legitimate users
7	0	It is possible for an attacker to browse the project yml file, especially if the repository is public. However, an attacker with access to the yml files can only view the workflow of the repository	Confirmation that an attacker can browse the projects yml files	Presence of trust boundary around the GitHub engine
8	0	To perform actions on GitHub, such as creating a pull request in a repository, a person must have sufficient access. The only way to gain push access is when the admin/owner of the repository assigns these permissions to you. Hence, an attacker can’t gain access if they are not directly assigned to them	Confirmation that attacker has push access only, which limits their functionality	Presence of a trust boundary between • user and local Git server• local Git server and remote server
9	0	An unauthenticated and non-privileged attacker can submit custom code into a remote repository, if vulnerabilities exists. However, changes made to code in a repository are often reviewed before being approved and merged to the master node. The approval process reduces the risk of unauthorized changes	Assumption that the attacker can reach the remote repository (e.g. through the Internet)	Presence of a trust boundary around remote Git server
10	0	GitHub uses HTTPS, which encrypts traffic between the server and client, which ultimately prevents unauthorized access. The HTTPS protocol provides an additional layer of security for users	Confirmation that GitHub uses HTTPS while the attacker managed to compromise HTTP	Presence of trust boundaries around local Git server, Remote Git server and GitHub actions

**Table 25:** Information needed to judge Validity of threats- Kubernetes scenario (Actual column, 1= actual threat, 0= fabricated threat)

ID	Actual	Justification	Textual hint	Additional hint (DFD)
1	1	In cases where the Kubernetes cluster is deployed in a public cloud (e.g., AKS in Azure, GKE in GCP, or EKS in AWS), compromised cloud credentials can lead to cluster takeover. Attackers with access to the cloud account credentials can access the cluster’s management layer	Confirmation that attacker can remotely interact with the server	Presence of data flow saving pod configurations in the etcD
2	1	This threat is possible by gaining access to a cluster using leaked credential configuration files, or by interacting with an unauthenticated API server of a cluster	Assumptions confirms a successful social engineering attack	Absence of a multi-factor authentication process
3	1	A container without CPU and memory resource limits can use up all the worker node’s resources (e.g., cryptocurrency mining scripts). This will likely cause crashing of other containers running on the same node, and in the worst case, the node itself	Confirmation that the attacker obtained a remote shell on a pod	Presence of pods and worker-nodes in the same trust boundary
4	1	Running a compromised image in a cluster can compromise the cluster. Attackers who get access to a private registry can plant their own compromised images. A user can then pull the latter. In addition, users often use untrusted images from public registries (such as Docker Hub) that may be malicious (supply-chain attacks)	•Scenario description detailing how a container image can be retrieved from a registry	Presence of image registry and image data store in the same trust boundary
			•Confirmation that attacker has permissions to upload or modify images in the registry used by the Kubernetes cluster	
5	1	Either by exploiting a vulnerability and getting a reverse shell, or by laterally moving to a privileged container, if an attacker gets a reverse shell on a privileged container, that is equivalent to having access to the container’s worker node, which means cluster take-over	• Scenario description of multiple containers being run inside a single pod	Presence of a trust boundary around the pod
			• Confirmation that attacker has access to privileged container	
6	0	The threat is fake because the network policies are implemented correctly, so no network traffic is allowed between the two namespaces	Confirmation that network policies are implemented correctly to segment the namespaces	Absence of container processes in different namespaces
7	0	An attacker with access to image A can only reverse image A and Dockerfile A, but not other images or Dockerfiles	Textual description of how a container image is retrieved from a registry	Presence of data flow to retrieve images where image name and tag are required
8	0	If an attacker compromises a running container, they can run any type of software/tool/process, independently from what is specified in the Dockerfile	Textual description of what is contained in a container (i.e. source code, libraries, and other dependencies)	Absence of security monitoring process
9	0	This threat may have been true if anonymous access was enabled in the API server. However, this is not specified, making it invalid because the attacker does not have enough privileges to upload custom configurations	No confirmation on whether anonymous access is enabled in the API server	Presence of trust boundary between user and master node containing the API server
10	0	A network bridge is a Layer 2 network divide. If the cluster is using a Layer 3 network plugin, the network bridge is not present, thus it can not be exploited	Scenario description that Layer 2’s virtual devices such as a network bridge are not usable in Layer 3	Absence of processes for several pods

**Table 26:** Technical knowledge needed to judge Validity of threats- GitHub scenario (Actual column, 1= actual threat, 0= fabricated threat)

ID	Actual	Justification	Technical knowledge
1	1	Using simple alert-style email notifications, attackers are able to steal credentials to gain access to development code, intellectual property, and project details	Knowledge on how social engineering attacks are carried out and the success factors
2	1	Git’s configuration files are plain-text, when leaked an attacker can view the personal information of the repository owner, leading to information disclosure attack. In addition, the attacker can alter the default bahaviour set by the owner by manually editing these values	Knowledge that Git configuration files are plain text and contains sensitive information such as usernames and email addresses
3	1	An attacker can exploit vulnerabilities (e.g., lack of authentication) in the git protocol to send multiple requests that overload or crash the server causing a DoS attack	Knowledge on • Exploiting authentication vulnerabilities (e.g., brute force attack on weak passwords, social engineering attacks)• How to perform a denial of service attack and its success factors
4	1	An attacker can get server and .../path/to/repo from git remote (only if the remote repository is of the ssh type). This permits the execution of server-side Git commands implementing the pull/push functionality	Knowledge that with the correct SSH keys the user can be authentication to the Git shell without requiring additional log in information)
5	1	With root access, the attacker is able to perform any actions on GitHub, such as creating a pull/push request in a repository	knowledge that only root access allows the execution of core git actions
6	0	It is possible for an attacker to submit a fake push request to other remote code repositories if they have access to the necessary credentials or exploit a vulnerability in the system. This could potentially cause a Denial of Service attack. But the assumptions clearly states that permission management is properly implemented	Knowledge on • Git’s permissions management• Success factors of a DoS attack
7	0	It is possible for an attacker to browse the project yml file, especially if the repository is public. However, an attacker with access to the yml files can only view the workflow of the repository	Knowledge that a YAML file contains human-readable data serialization used to define workflows and configuration. They are not executable.
8	0	To perform actions on GitHub, such as creating a pull request in a repository, a person must have sufficient access. The only way to gain push access is when the admin/owner of the repository assigns these permissions to you. Hence, an attacker can’t gain access if they are not directly assigned to them	Knowledge of Git’s permissions management which controls a user’s access to a repository through the assigned roles
9	0	An unauthenticated and non-privileged attacker can submit custom code into a remote repository, if vulnerabilities exists. However, changes made to code in a repository are often reviewed before being approved and merged to the master node. The approval process reduces the risk of unauthorized changes	Knowledge of Git’s version control alongside pull request approval process
10	0	GitHub uses HTTPS, which encrypts traffic between the server and client, which ultimately prevents unauthorized access. The HTTPS protocol provides an additional layer of security for users	Knowledge that HTTPS offers an additional security layer, which encrypts communication

**Table 27:** Technical knowledge needed to judge Validity of threats- Kubernetes scenario (Actual column, 1= actual threat, 0= fabricated threat)

ID	Actual	Justification	Technical knowledge
1	1	In cases where the Kubernetes cluster is deployed in a public cloud (e.g., AKS in Azure, GKE in GCP, or EKS in AWS), compromised cloud credentials can lead to cluster takeover. Attackers with access to the cloud account credentials can access the cluster’s management layer	Knowledge that exploiting authentication vulnerabilities and gaining valid credentials can lead to a cluster take-over
2	1	This threat is possible by gaining access to a cluster using leaked credential configuration files, or by interacting with an unauthenticated API server of a cluster	Knowledge on how social engineering attacks are carried out and the success factors
3	1	A container without CPU and memory resource limits can use up all the worker node’s resources (e.g., cryptocurrency mining scripts). This will likely cause crashing of other containers running on the same node, and in the worst case, the node itself	Knowledge that a container has no resource constraints, by default. However, mitigations such as setting runtime configuration
4	1	Running a compromised image in a cluster can compromise the cluster. Attackers who get access to a private registry can plant their own compromised images. A user can then pull the latter. In addition, users often use untrusted images from public registries (such as Docker Hub) that may be malicious (supply-chain attacks)	Knowledge that a cluster’s access can be set as public or private
5	1	Either by exploiting a vulnerability and getting a reverse shell, or by laterally moving to a privileged container, if an attacker gets a reverse shell on a privileged container, that is equivalent to having access to the container’s worker node, which means cluster take-over	Knowledge of lateral movement between containers
6	0	The threat is fake because the network policies are implemented correctly, so no network traffic is allowed between the two namespaces	Knowledge on the benefits of using namespaces; isolate resources, define access controls, e.t.c
7	0	An attacker with access to image A can only reverse image A and Dockerfile A, but not other images or Dockerfiles	Knowledge that one can only reverse engineer the images one has access to. Images are typically stored in separate filesystems and registries, and they are isolated from one another at runtime
8	0	If an attacker compromises a running container, they can run any type of software/tool/process, independently from what is specified in the Dockerfile	Knowledge that with the right permissions (root access) to the container, an attacker can download and run additional tools, install software, or modify existing files
9	0	This threat may have been true if anonymous access was enabled in the API server. However, this is not specified, making it invalid because the attacker does not have enough privileges to upload custom configurations	Knowledge that unauthenticated users can send API requests to the Kubernetes API server only if anonymous access is enabled
10	0	A network bridge is a Layer 2 network divide. If the cluster is using a Layer 3 network plugin, the network bridge is not present, thus it can not be exploited	Knowledge of the differences between Layer 2 and Layer 3 of the OSI model

### A.4 Prompt Template

As discussed in Subsection 5.6, participants in Groups A and C were provided with the relevant fields for the body of the prompt for each threat, which included a threat description, assumption, STRIDE threat type, and affected components. Participants were then allowed the freedom to interact with the LLM.

Table 28 provides an instance of the body of the prompt for each scenario. For the full material provided to the participants, we invite the reader to refer to the replication package (Bahati Mbaka andTuma 2025).

**Table 28:** Prompt template for one of the threats in the two scenario descriptions

Scenario	Updating a remote repository on GitHub
Threat description	Spoofing a remote repository by stealing the authentication credentials of the admin via a social engineering attack
Assumptions	The attacker carries out a successful social engineering attack (attackers communicate legitimately with others, manipulating and exploiting human qualities to achieve their attack) and gets authentication credentials. The credentials are valid
STRIDE threat type	Spoofing
Affected components	The remote code repository
Scenario	Deploying a pod on Kubernetes
Threat description	If an attacker compromises a pod in a Kubernetes cluster using a Layer 3 network solution, he/she can steal other pods’ identities and laterally move within the cluster using the network bridge
Assumptions	The attacker exploits one container, The CNI works at Layer 3
STRIDE threat type	Spoofing
Affected components	Lateral movement between pods

## Footnotes

1 https://attack.mitre.org

2 The training delivered to groups A and B also included a DFD of the scenario.

3 Where point 1 is labeled "strongly disagree", point 3 is labeled "neutral", and point 5 is labeled "strongly agree".

4 https://atlasti.com/

5 www.qualtrics.com/
